# Application of LCA Method for Assessment of Environmental Impacts of a Polylactide (PLA) Bottle Shaping

**DOI:** 10.3390/polym12020388

**Published:** 2020-02-09

**Authors:** Patrycja Bałdowska-Witos, Weronika Kruszelnicka, Robert Kasner, Andrzej Tomporowski, Józef Flizikowski, Zbigniew Kłos, Katarzyna Piotrowska, Katarzyna Markowska

**Affiliations:** 1 Department of Technical Systems Engineering, Faculty of Mechanical Engineering, University of Science and Technology in Bydgoszcz, 85-796 Bydgoszcz, Poland; weronika.kruszelnicka@utp.edu.pl (W.K.); robert.kasner@gmail.com (R.K.); a.tomporowski@utp.edu.pl (A.T.); fliz@utp.edu.pl (J.F.); 2 Institute of Machines and Motor Vehicles, Faculty of Transport Engineering, Poznan University of Technology, 60-965 Poznan, Poland; zbigniew.klos@put.poznan.pl; 3 Faculty of Mechanical Engineering, Lublin University of Technology, 20-618 Lublin, Poland; k.piotrowska@pollub.pl; 4 Department of Logistics and Transport Technologies, Faculty of Transport and Aviation Engineering, Silesian University of Technology, 40-019 Katowice, Poland; Katarzyna.Markowska@polsl.pl

**Keywords:** PLA bottle, bio-based and biodegradable polymers, life cycle assessment, environmental impact, ReCiPe2016 method

## Abstract

In recent years, there has been a significant increase in the consumption of single-use packaging. Their material diversity is a significant barrier to recycling, causing overloading of landfills. Increasing negative environmental aspects have highlighted the need to develop solutions to achieve a relatively high efficiency of the bottle shaping process with the lowest possible energy consumption. The aim of the project is to try to describe the impact of this process on the state, transformation and development of the natural environment. The work concerns current issues of the impact of packaging on the natural environment. The main goal was to conduct a life cycle analysis (LCA) of beverage bottles made of polylactide. The functional unit comprised a total of 1000 pieces of PLA bottles with a capacity of 1 L. The boundary of the adopted system included the steps from the delivery of the preforms to the production plant to their correct formation in the process of forming beverage bottles. Further stages of the production process were excluded from the system, such as beverage bottling, labeling, and storage and distribution. Processes related to transport and storage of raw material were also excluded. The LCA analysis was performed using the program of the Dutch company Pre Consultants called SimaPro 8.4.0. The ReCiPe 2016 method was chosen for the interpretation of the quantity of emitted substances into the natural environment. The test results were presented graphically on bar charts and subjected to verification and interpretation.

## 1. Introduction

The commercialization of biodegradable materials under industrial composting in place of the non-degradable polymer materials currently used is a technologically and socially complex process [1]. Technological progress in the field of biodegradable polymers as well as consumer expectations motivate producers to undertake actions to gradually replace the fossil fuel-based sources with natural sources for polymer production [2,3].

Nowadays, environmentally conscious producers not only produce their products from partially or completely recycled materials, but they are also increasingly using biopolymers in addition to or instead of petroleum-based polymers. Like petroleum-based plastics, most biopolymers are used by the packaging industry [4]. However, due to their function, they have a very short life span (on average a few weeks), which is why they become a waste in a short time [5,6]. In 2016, 16.7 million tonnes of plastic packaging waste was collected, of which 40.8% was recycled, 38.8% was used to generate energy (combustion), and 20.4% went to landfills [7].

In May 2019, the Council of the European Union proposed new EU-wide rules for 10 single-use plastic products that are most often found in Europe’s seas and beaches. Member States must, no later than two years after the entry into force of the Directive, prohibit the following single-use plastic products: plastic swabs, cutlery, plates, straws, stirrers and balloon sticks; all products made of oxo-plastic; and cups, food and beverage containers made of foam. In addition, 90% of single-use plastic bottles should be collected separately by 2029 [8].

In 2017, only about 2% of total plastic production was biopolymer, but its volume is growing every year [9]. With the entry into force of the directive, this increase is likely to be even greater. Poly (lactic acid) (PLA) is one of the most popular biodegradable biopolymers used in the packaging industry for the production of films, sheets, bottles and foams, and its growing use in industry intends to replace the currently used forms of polymeric polyethylene terephthalate (PET) [10,11]. Considering the above, it was considered necessary and possible to conduct an experiment to evaluate the cycle of the process of shaping biodegradable plastic bottles in an industrial composting plant [12,13].

The analysis of many works on the impact of the life cycle of products such as disposable bottles on the life cycle indicates that it is necessary to identify some sets of impacts and their hierarchy. At the same time, it is important to remember what environmental costs have already been incurred and what costs will probably be generated in the future. There is also a relationship between interactions and design solutions of the analyzed bottle, e.g., by using fillers or compatibilizers.

Using the life cycle analysis (LCA) method, companies declare a reduction of environmental impact to a greater extent than the competition, and the results of the assessment shape new production directions, taking into account such factors as: sources of materials, suitability for recycling and use of recycled materials, reduction of greenhouse gas emission rates. In the literature, you can often find the results of analyses of environmental impacts of various objects, production processes and packaging. The vast majority of these analyses are based on the concept of life cycle analysis. Analyzing the data of these analyses, it can be seen that the greatest environmental nuisance is the stage of operation of technical facilities. Among the results of analyses published by N. Horowitz, J. Frago, D. Mu (2018) [14] regarding the life cycle assessment of bottles made of PLA or PET, it is possible to determine potential levels of impact on the state of the environment. However, the presented results show only impact levels for the entire technological process of bottle production. In turn, the results presented by L. Chen, R. Pelton, T. Smith (2016) [15] illustrated the potential level of inflows for bottles produced in PET and PET obtained in 100% from the raw material subjected to the recycling process. Additionally, these studies did not refer to the estimation of the potential of levels of impacts of individual unit processes at the time of bottle formation. A similar approach was presented in the research of S. Papong, P. Malakul, R. Trungkavashirakun, P. Wenunun, T. Chomin, M. Nithitanakul, E. Sarobol (2014) [16], which focused on demonstrating the potential environmental impacts of the water bottle production process drinking from polylactic acid produced in Thailand. Analyzing the results of literature studies, no studies could be found regarding the assessment of potential impacts of the process of shaping beverage bottles [17,18]. Due to some limitations and a lack of data availability, most studies focus on analyzing cradle-to-grave processes. The reason for these limitations is primarily the varied means of operating machines and devices, which would require diversity and a multi-faceted approach to diagnose all impacts.

## 2. Materials and Methods 

The methodology of environmental assessment of an object life cycle was developed in the 1990s and was formalized by introduction of norm of ISO 140040-43 series, which defines four stages of the assessment: selection of a functional unit, the system boundaries; analysis of the set of entries and exits; transformation of collected data into indexes of impact categories or damage categories and conclusions and verification of results [19].

The ReCiPe method, whose abbreviation stands for the institutions which were involved in its creation, that is: National Institute for Public Health and the Environment (RIVM) and Radboud University Nijmegen, CML (in Dutch: Centrum voor Milieuwetenschappen, in English: Institute of Environmental Sciences of the University of Leiden) and PRé, is the result of conversion of a long list of the life cycle inventory results into a limited number of indexes defined for two impact and damage category levels [20].

This method determines the middle and the final points, but also includes factors consistent with three cultural perspectives [21,22]. These perspectives make up a set of choices on issues such as time or expectations, which can provide the basis for appropriate management or future technological development in order to prevent from damaging the natural environment or reduce it as much as possible. 

Contrary to other precisely described methods, such as Eco-Indicator 99, Environmental Priority Strategies (EPS) or IMPACT 2002+, indicator ReCiPe 2016 does not cover the potential future impacts for the category of damage assuming that such impacts have been taken into account in the analysis of inventory. Other differences can be observed in its approach to the category of damage which involves ‘exhaustion of natural resources’. 

The IMPACT 2002+ method accepts the amount of primary energy in megajoules (MJ) to be a unit, whereas an increase in fuel extraction costs is a unit of the ReCiPe method (in dollars) [23].

**Figure 1:**
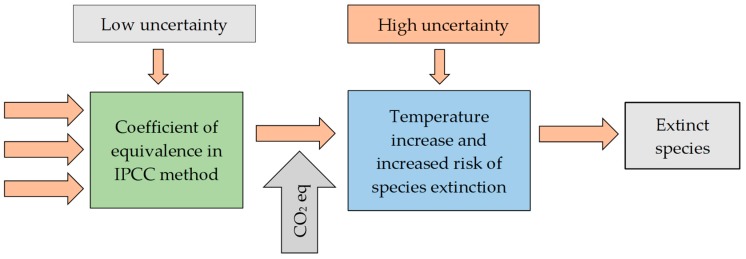
An example of a harmonized model of the final point regarding climate change connecting human health and damage to ecosystem.

Moreover, ReCiPe 2016 uses an environmental mechanism which can be viewed as a series of results which when combined can cause a certain level of damage, for instance to human health or ecosystems. In result of the climate change, many substances increase their emissions which means that heat does not escape from the earth into space. In consequence, more energy is being trapped on earth and the temperature increases. This contributes to changes in the natural habitats of living organisms, which can lead to the extinction of some species of animals and plants [23]. Figure 1 shows that the longer it takes for the environmental mechanism to be formed, the higher the uncertainties. The temperature increase is more difficult to determine because there are many parallel positive and negative feedbacks [23,24].

The ReCiPe 2016 method involves performing calculations according to the specified eighteen indexes of the middle point and three indexes of the final points [25]. Since interpretation of a large number of indexes of the middle point is difficult, there is a motivation for calculation of indexes of the final point. Application of the final point level indexes is supposed to facilitate interpretation of the results because they represent impacts of the analyzed processes or products, only for three areas: human health, ecosystem quality, and climate changes [23]. 

### 2.1. Goal and a Functional Unit

The research goal is characterized by the research specificity, insight and scope and the types of data needed for the life cycle assessment. LCA is a tool for assessment of the product general impact on the natural environment “from the cradle to the grave”. The choice of the process which is least harmful in terms of human health and natural environment is a priority. For this purpose, a detailed assessment of biodegradable polymer bottles shaping process was performed at the stage of characterizing. The major goal of this study is identification of the areas with potentially the highest harmful environmental impact. The basic assessment of the life cycle was limited to selected impact categories: global warming, formation of fine particles, use of water resources, water acidification and land use. Calculations were performed based on an Ecoinvent version 3.3, database library implemented in the SimaPro 8.4.1, program. One thousand bottles of 1 L volume were accepted to be a functional unit.

### 2.2. The System Boundary 

Six separate unit operations consistent with the process accepted for production of bottles were assumed to be the system boundary. These include: preheating of the preform (PBH), heating the preform (PH), stretching and extension of the preform (PSE), pressure shaping of the preform (PPS), degasification (DB) cooling of the shaped bottle (CB) [23]. Adopting the same assumptions based on an identical division of unit operations as in the work [23] enables comparison of two processes of shaping bottles made of PET and PLA. The presented analyses of technological processes in the production of bottles for beverages [23] are characterized by diverse potential impact on the state of the natural environment and depend on the amount of electricity consumed in the production process. The high level of impact on human health is determined by the poly (ethylene terephthalate) used in the production process. To reduce the negative impact on the state of the natural environment, food industry producers should constantly seek substitutes for raw materials from exhaustible fossil resources for which the ideal replacement is biodegradable in an industrial composting facility. The right direction of development is to popularize biodegradable raw materials of natural origin. As a result, the technological operations of the accepted processes were burdened with the same simplifications which made it possible for each of the considered technological operation to accept the level of exclusion to be below 0.01% of the share in the whole life cycle and in all potential environmental impacts. The collected data cover the production, use of resources, energy consumption, emissions to the air, soil and water (Figure S1, Table S1).

**Figure 2:**
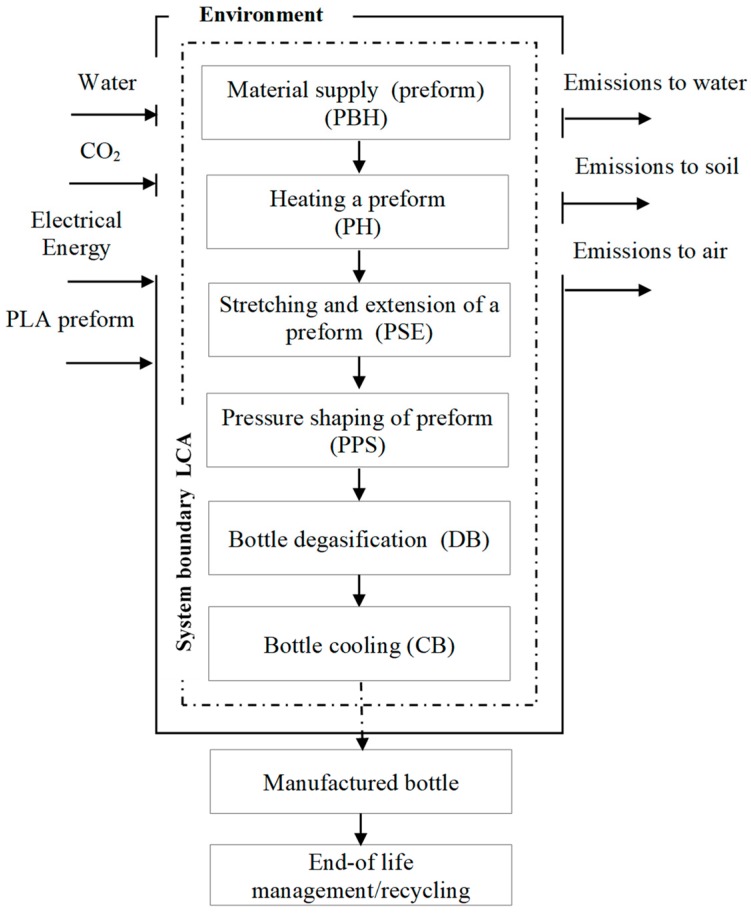
Boundaries of the accepted system [23].

The first stage of the research involved checking sufficiency and consistence of the accepted measurement data. Due to the character of the research which focused on assessment of the bottle shaping processes, whereas the elements related to the material delivery to the production plant and further stages of the product use were excluded (Figure 2).

## 3. Results

**Figure 3:**
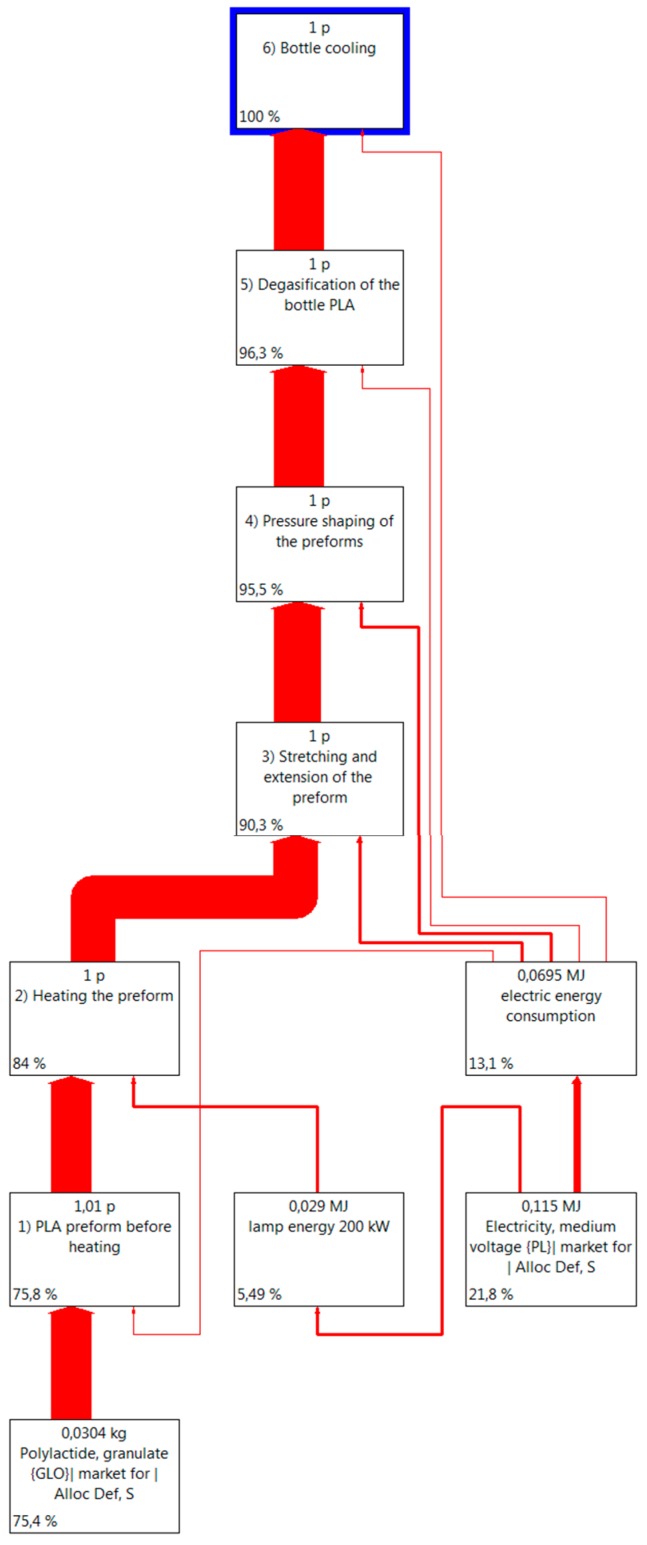
Tree depicts the processes involved in a PLA bottle shaping PLA.

Taking into account the confidential character of the results presented in the study and company trade secrets, the values presented in Table 1 were changed by a coefficient ranging from 0.8 to 1.2 [20,24,25,26]. A tree of the processes depicting the flow of materials and energy during the whole life cycle of the analyzed object was developed. The tree, based on the above given assumptions of the beverage PLA bottle production process, is shown in Figure 3 while maintaining a 0.99% abscission level. This level covers all the process ranges including materials and media.

## 4. Analysis of Test Results

The next stage of the analysis involved correct estimation of potential environmental impacts obtained at the stage of characterizing. The analysis results are included in Table 2. The results are presented in three basic units characteristic of the ReCiPe2016 method: Disability Adjusted Life Years (DALY)—life years of disability, Species Year (species.yr)—time-integrated loss of local species, Dollar ($)—additional costs connected with future extraction of fossil fuels and minerals [23,24,25,27,28,29].

Greenhouse gases (GHG) that contribute to global warming [26], such as CO_2_ and methane, are often released to the atmosphere by natural causes (that is volcanoes and breathing) of anthropogenic origin (that is, from chimneys, motor vehicles, stoves of apartment buildings, factories) [23]. The process of a properly shaped product degasification was reported to have the highest potential negative environmental impact on human health (1.16347 × 10^−8^ DALY), on land ecosystems (3.51106 × 10^−11^ species.yr) and fresh water ecosystems (9.5902 × 10^−16^ species.yr), with the material total life cycle contribution being about 78%, for all the three impact categories. 

Carcinogenic toxicity for people and carcinogenic factors being non-carcinogenic or respiratory substances are the environmental effects. It has been proven that most carcinogenic substances PLA (2.59368 × 10^−10^ DALY) are released before the preform is supplied to a furnace for heating. However, the greatest potential effect on carcinogens for PET bottles was recorded for the entire production process with a value of (87.6%) [23]. The emissions of nickel and cadmium ions were found to have the most damaging effect in both of the considered processes. The highest potentially harmful impact was found for a shaped freshwater ecotoxicityPLA (3.0593 × 10^−12^ DALY) bottle during the process of cooling. This is caused by the fact that production of resin PET—oil derivative—is usually accompanied by a release of cancerogenic substances in the process of the material manufacturing (Figure S2, Table S2), (Figure S3, Table S3), (Figure S4, Table S4). 

**Figure 4:**
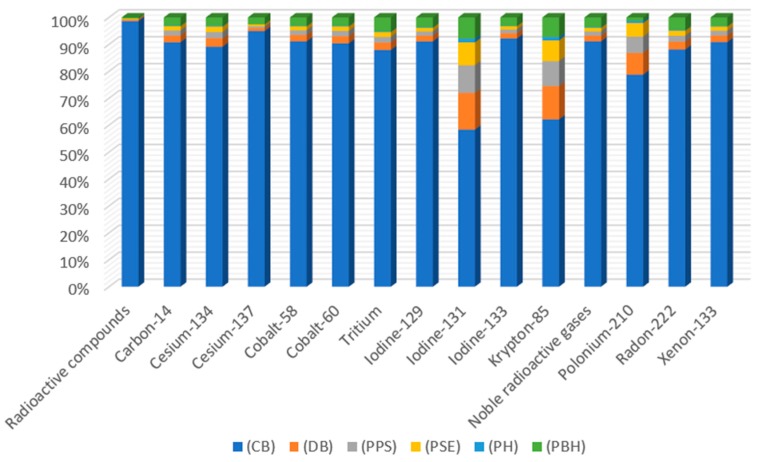
The results of characterization of the amount of ionizing radiation emissions into the air throughout the entire cycle of shaping bottles for beverages made of PLA, which is biodegradable in an industrial composting facility.

In the entire life cycle of beverage bottles, among all compounds in the analyzed category, the highest level of negative emissions to air is characterized by the radon isotope—^222^Rn (Table 3, Figure 4). Most of this noble gas with high ionizing capacity is generated at the stage of cooling the 1.45 × 10^−12^ DALY bottle. The main source of emissions of this compound is the raw material and the amount of refrigerant used. 

**Figure 5:**
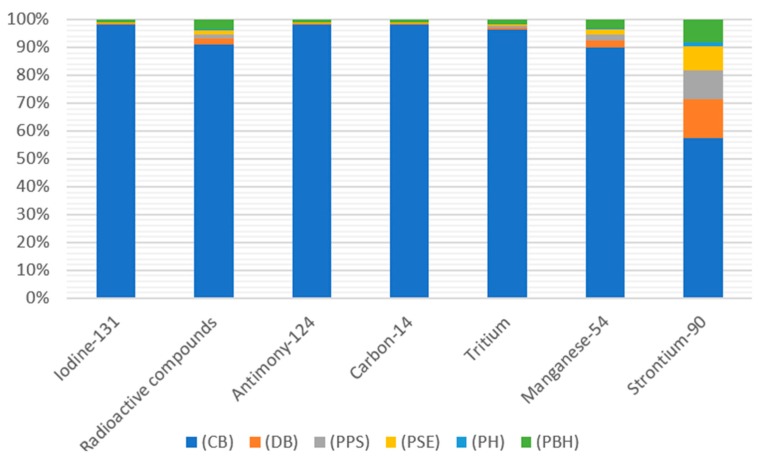
The results of characterizing the amount of ionizing radiation compounds emitted to water throughout the entire cycle of shaping bottles for beverages made of PLA which is biodegradable in an industrial composting facility.

When analyzing the harmfulness of compounds emitted to water, the greatest pollution was recorded for Tritium at the stage of cooling the finished bottle (Table 4, Figure 5). The main source of environmental harm is the amount of refrigerant used and the raw material.

The main sources of water pollution depend on the usability and innovation of the technological line, which has limited possibilities of reducing pollution as part of the production process. New installations have greater opportunities to adapt to environmental requirements through the use of technologies to prevent specific waste groups. The presented production process has a specific type of waste that can be determined knowing the characteristics of: construction materials, corrosion and erosion mechanisms, and consumables. Waste prevention includes: minimization of waste generation and recycling.

Non-carcinogenic toxicity for people had the highest potential impact for a bottle produced from PLA (9.21497 × 10^−11^ DALY). Emissions of non-cancerogenic compounds were found to be the highest at the stage of PLA bottle degasification, whereas the second highest value in terms of emission was the process of a bottle pressure shaping (7.04032 × 10^−11^ DALY) and the process of automatic stretching and extension of a pre heated preform PLA (5.71376 × 10^−11^ DALY). The total share of the PLA material in the process of technological shaping of a PLA bottle was merely (1.76 × 10^−9^ DALY); it exhibits almost four times lower potential environmental impact than a bottle produced from a non-biodegradable material. The source of electrical energy is largely responsible for emissions of non-cancerogenic substances and the amount of their emission gradually grows along with the progress of the production process. Degasification of a shaped PLA bottle is very fast and consumes a large amount of electric energy in a very short time, which leads to an increase in potential impacts on the noncarcinogenic substances.

Ultraviolet radiation can destroy organic matter [23]. Some compounds decompose the ozone layer, particularly derivatives of methane and ethane containing atoms of chlorine, chlorine and fluorine and bromine and fluorine [23]. In the case of PLA bottle shaping, the value (1.61 × 10^−13^ DALY) was reported while for the PET bottle shaping process a lower value was recorded (1.66 × 10^−11^ DALY) [23]. Similar values were found for unit processes for which the highest negative impact was reported for the bottle degasification process, whereas the lowest for the category of bottle cooling. The basic stage responsible for high value is the penultimate stage of bottle production, characterized by values of 97.81% of the impact for PLA bottles and 80.58% for a regular PET bottle [23].

Ecotoxicity of the aquatic and land environments is caused by poisonous toxic substances which are released to the natural environment [23]. Marine ecotoxicity has potentially the highest negative impact on the aquatic environment (Figure S5, Table S5) The highest similar emission levels were recorded for the degassing process of the shaped PLA bottle (7.1106 × 10^−16^ species.yr) and PET (6.97002 × 10^−16^ species.yr). Similar emission values were also found for both processes in which the PET bottle shaping process contributed to the ecotoxicity of the aquatic environment in 92.65%. While PLA bottles in 90.75% (Figure 4, Table 4) [23]. 

Freshwater ecotoxicity was featured by the highest potential value of emission defined for the process of PLA perform heating (1.04682 × 10^−15^ species.yr), second in terms of the emission of negative compounds to the aquatic environment was the same unit process describing the PET preform (1.01331 × 10^−15^ species.yr) [23].

The terrestrial ecotoxicity was highest affected by the PET bottle production process (4.95 × 10^−12^ species.yr) [23]. Among the six unit processes analyzed, the smallest negative impact of the shaped bottle cooling process was recorded for the PLA bottle (5.31222 × 10^−15^ species.yr). Comparing two bottle formation processes, it can be stated that the highest level of negative impact on terrestrial ecotoxicity was characterized by the raw material used for the PET bottle shaping process, as much as 92.97% [23], while the raw material used for the PLA bottle shaping process affected terrestrial ecotoxicity in 88.26% (Figure S6, Table S6).

Land environment acidification is caused by a decrease in the pH value. This phenomenon occurs as a result of disturbing the ecological balance of energy and matter exchange processes between elements of ecosystems. The reason for such changes is the presence of chemical substances. Regular land acidification occurs when gases such as CO_2_ or SO_2_, are absorbed by water and react to form acidic compounds on the surface of the earth [26,27,28,29,30]. The magnitude of the impact of the process of shaping bottles from biodegradable material (1.46 × 10^−10^ species.yr) was greater than that of shaping PET bottles (1.14 × 10^−10^ species.yr). The impact of individual raw materials in the bottle shaping process was 76.00% for PLA and 72.32% for PET, respectively. The smallest negative impact in the entire shaping process belonged to the cooling process of the finished product, while the largest degassing process of the bottle [23]. 

Land use was definitely higher for the production of corn-based bottles. This is confirmed by the fact that in the whole process of shaping the bottle, as much as 95.35% of the revenues of all categories is the raw material used for production. Characterizing the entire process of a PLA bottle shaping it needs to be noted that the highest potential influence on the land use was found for the process of a ready bottle degasification (1.73%), slightly lower influence (1.32%) was found for the process of the perform pressure shaping, nearly 1% impact was observed for the process of the preform stretching and extension in a mold, whereas, below 1%—for the preform processes applied before heating and cooling of a ready product. 

The contribution of polylactide. being as much as 97.87% was found for the water eutrophication. This phenomenon is well, understood as cultivation of corn requires application of fertilizers which adversely affect the natural environment. 

Ionizing radiation is a phenomenon which has always been present in the environment [31]. Its main source is radon emitted from the earth’s crust [32]. The ubiquity of radioactive elements in nature makes humans a source of radiation as well [33]. The highest potentially negative environmental impact was involved in one of the sub-processes and depended on the amount of a semi product—PLA perform used in the process. The value of emissions was found to be (2.11 × 10^−13^ DALY). The PET preform 2.00518 × 10^−13^ DALY showed a slightly lower negative value. Relatively similar values were recorded for two raw materials used in the process of shaping PET bottles (88%) [23] and PLA (89%). 

Particulate matter is generated during the process of bottle shaping by engines of the blowing machine. Fine particles referred to as PM (particulate matter), include those components of the bottle production process whose state when leaving the engine is different from gaseous. The most harmful substances accompanying formation of fine particles are PAH—polycyclic aromatic hydrocarbons [30]. Hence. based on the analysis results, the highest negative impact of the bottle shaping process on human health was found for the process of a PET bottle creation (1.17629 × 10^−08^ DALY) at the stage of degasification of a ready product. The highest value of negative emissions of particulate matter on human health was reported for the process of a PET bottle cooling (5.44468 × 10^−10^ DALY). 

Water use has an influence on human health and the quality of land and aquatic ecosystems. Water is of key importance for the global industry. A bottle production plant uses significant amounts of raw material. that is, corn starch. The production material has an impact on the natural environment. For each of the three analyzed impact categories it was the process of PLA bottle formation that caused potentially the highest damage to human health and the quality of aquatic and land ecosystems (6.8291 × 10^−9^ DALY; 4.15283 × 10^−11^ species.yr; 1.85801 × 10^−15^ species.yr). 

The PET bottle had the greatest potential impact on the mineral and fossil resource category. The process of shaping it showed the highest potential level of unfavorable influences in the process of collecting preforms for the heating furnace (2.71165 × 10^−6^ USD2013) for the category of mineral resource deficiency (Figure S7. Table S7). With the growing global demand for mineral resources, it is important to analyze whether the resources of geologically and technically available minerals in the earth’s crust can meet the future needs of humanity. Widespread recycling, increasing material efficiency and demand management will certainly play an important role in satisfying future generations. A significant high value of potential inflows was recorded for the category of scarcity of fossil resources. The bottle with the greatest impact on this category was the PET bottle (8.58689 × 10^−5^ USD2013) [23] in the process of stretching and lengthening the preform, the process of collecting preforms for the heating furnace (7.00854 × 10^−5^ USD2013) was responsible for slightly less negative impact. This phenomenon is related to the fact that PET requires continuous extraction of fossil fuels, resulting in their depletion. This behavior confirmed the highest stage impact. which is bottle production, and therefore probably the PET bottle had the greatest impact in the category of mineral extraction. Bottle production had a slightly smaller impact in this category, probably due to the fact that extraction of resources is also necessary. 

**Figure 6:**
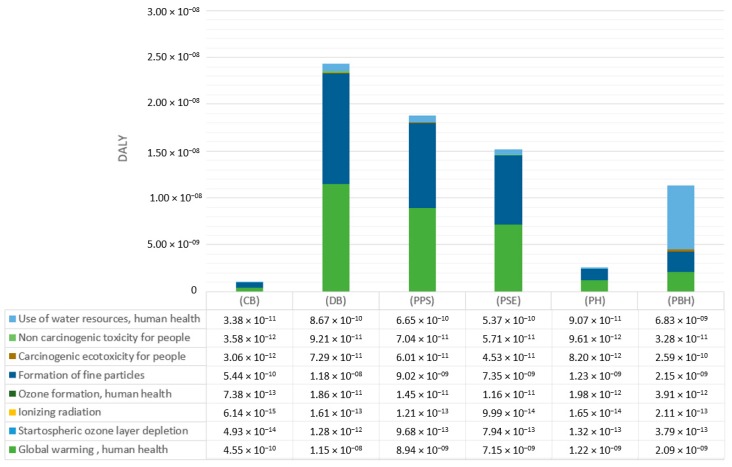
Results of characterization of environmental impacts for 8 impact categories in terms of human health.

Out of all the discussed categories, those that were chosen were those whose summary life time impact involved in a biodegradable bottle shaping was 90%. Selected results of the characterizing of the environmental impacts for each stage of the bottle shaping process in terms of human health is presented in Figure 6. 

The highest levels of negative impact on human health were found for: global warming (1.27 × 10^−7^ DALY), particulate matter formation (1.61 × 10^−7^ DALY), and use of water resources (2.93 × 10^−8^ DALY), which is caused by the use of a material—polylactide—as well as by emitting compounds to the natural environment during the process of bottle shaping. 

**Figure 7:**
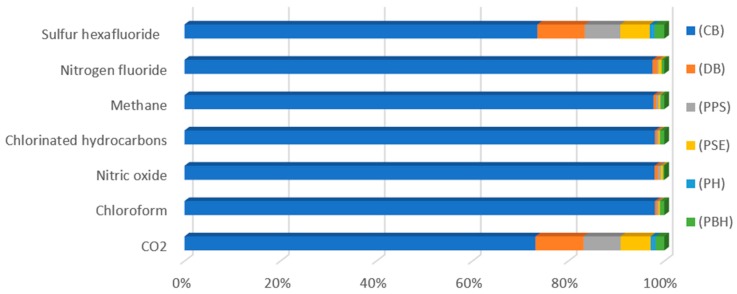
Results of characterization of environmental impacts for the category of global warming, (DALY).

The highest negative impact on human health was on the part of the global warming category emissions (Table 5, Figure 7) caused by emission of the compounds carbon dioxide (7.68 × 10^−8^ DALY) and nitric oxides (7.48 × 10^−9^ DALY) at the stage of end product cooling, which is connected with the process of bottle shaping and its given parameters. 

**Figure 8:**
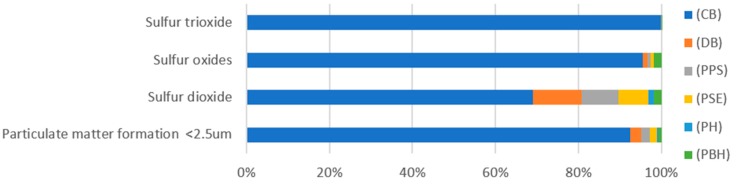
Results of environmental impact characterization for the category of particulate matter formation (DALY).

The most detrimental effect on human health involved in the entire bottle shaping life cycle was found for the groups of particulate matter (Table 6, Figure 8), of which the largest impacts were caused by formation of fine particles < 2.5 um (7.13 × 10^−8^ DALY) and emission of sulfur dioxide (5.75 × 10^−8^ DALY) at the stage of the end product cooling. The least potentially harmful impact on human health and the environment was observed for emission of SO_3_ (5.32 × 10^−18^ DALY) at the stage of the preform heating.

**Figure 9:**
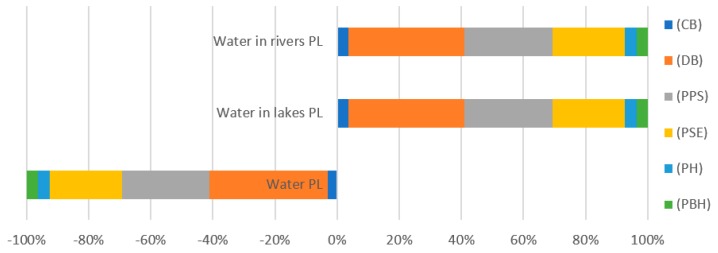
Results of environmental impact characterization for the category of water use (DALY).

The results of environmental impact characterization for the category of water consumption, which is a medium (Table 7, Figure 9) used for the process of bottle production, show that the highest positive impact was found for the water used at the stage of a ready bottle degasification and cooling. This is due to the fact that the water used in the production process circulates in a closed system and its amount and control frequency is adjusted to the production size and volume and other respective parameters. 

**Figure 10:**
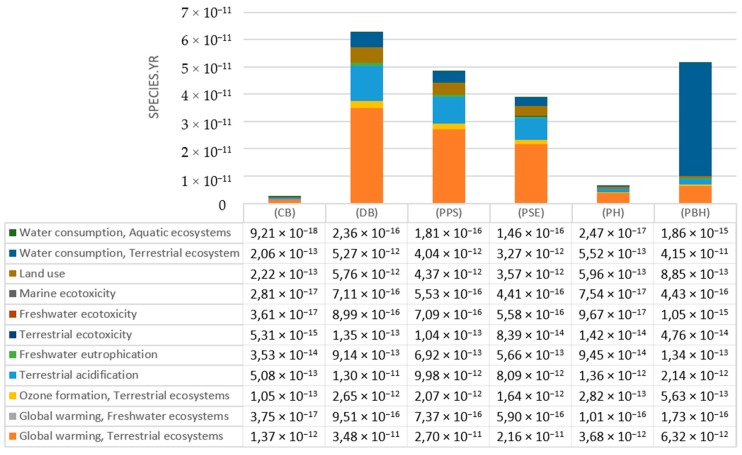
Results of environmental impact characterization for 11 categories of ecosystem shaping.

Certain impact categories were particularly harmful to the natural environment throughout the life cycle of the bottle shaping process (Figure 10), i.e., global warming (1.04 × 10^−14^ species.yr), land acidification (1.47 × 10^−10^ species.yr), and land use (3.31 × 10^−10^ species.yr) contributing to climate changes. The lowest negative impacts were observed for the remaining categories, including land and marine toxicity, ozone layer creation, fresh water eutrophication and global warming affecting the fresh water ecosystems (Figure S8, Table S8), (Figure S9, Table S9), (Figure S10, Table S10), (Figure S11, Table S11), (Figure S12, Table S12). 

**Figure 11:**
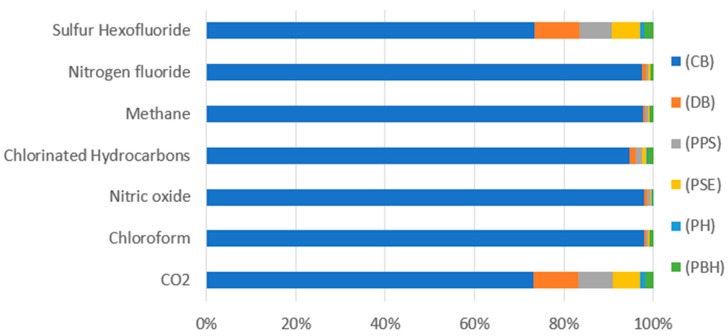
Results of environmental impact characterization for the category of global warming (species.yr).

In the case of the first category (global warming), featuring potentially the highest negative impact on the ecosystem (Table 8, Figure 11), just like in the category of human health, the same groups of components, although with different emission values. were observed: CO_2_ (2.3162 × 10^−10^ species.yr), nitric oxide (2.2508 × 10^−11^ species.yr). A comparison of the same compounds but different damage categories shows that both CO_2_ and NO_x_ featured higher negative effect on human health in the process of polylactide bottle shaping.

**Figure 12:**
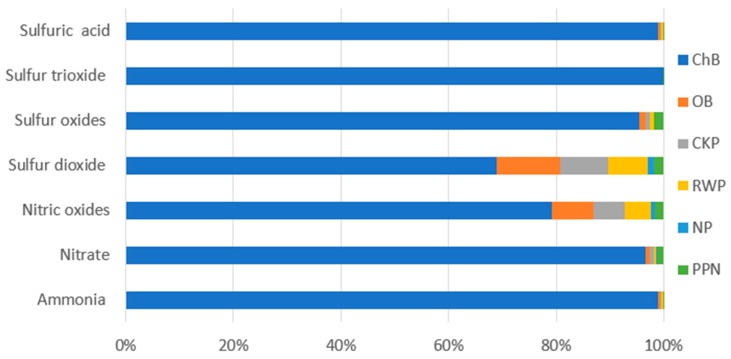
Results of environmental impact characterization for the category of land acidification (species.yr).

The stage of a ready bottle cooling (Table 9, Figure 12) featured the highest summary emissions of compounds into the atmosphere. At this stage. the highest levels of emissions were observed for sulfur dioxide (6.7 × 10^−11^ species.yr), sulfur oxides (3.59 × 10^−16^ species.yr), sulfuric acid (2.88 × 10^−11^ species.yr) and nitric oxide (1.6 × 10^−11^ species.yr), which are generated as a result of electric energy use in the process of a bottle manufacturing. At this stage, each reduction of the device working parameters to decrease electric energy consumption, contributes to atmospheric emission reduction. 

**Figure 13:**
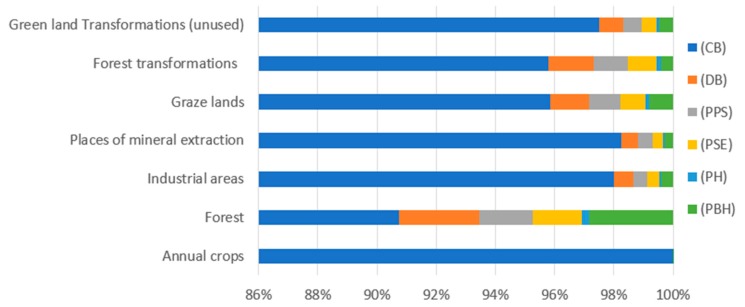
Results of environmental impact characterization for the category of land use (species.yr).

The results of characterizing of environmental impacts of the processes connected with land use involved in a biodegradable bottle production process life cycle are presented in Table 10 and Figure 13. Of all the considered processes of the analyzed impact category. The highest level of negative environmental impact was reported for the process of agricultural cultivation whose value at the last stage was (2.64 × 10^−10^ species.yr). Such a big difference in particular levels of emission is caused by increased use of land for cultivation of corn for the production of corn starch. 

**Figure 14:**
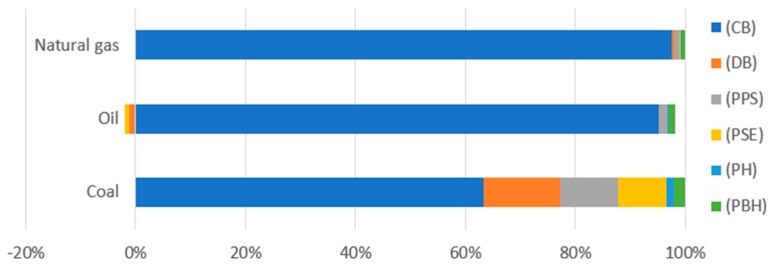
Results of environmental impact characterization for the category of fossil fuel depletion (USD2013).

The last step of the analysis was to estimate the environmental impacts associated with exhaustion of fossil fuels (Table 11, Figure 14). According to the analysis results. the highest level of harmful environmental impact was found for the process of coal and natural gas extraction. at each stage of a bottle production. Emissions involved in the processes of oil extraction were observed to be positive at the stage of stretching of the preform and during degasification of the product. 

## 5. Conclusions

The results presented in this study clearly indicate the emission of certain groups of components. that is, carbon dioxide, nitric oxides and sulfur oxides into the natural environment. This is caused by the use of raw materials, electrical energy and water throughout the life cycle of polylactide bottle shaping. 

The results of this study show that the processes of end product degasification and cooling (in terms of used materials and electrical energy consumption) have the highest contribution in emissions of CO_2_, nitric oxides, sulfur oxides and formation of fine particles of below 2.5 um. 

In view of the obtained results, it needs to be said that in order to improve the natural environment, it is necessary to use methods for reduction of the perform mass as early as at the stage of the bottle shaping. The consumption of electrical energy should be reduced as well.

In result of the research it was assumed that for further analysis of the environmental impacts it is necessary to carry out an analysis of the quality of point data and uncertainty of results.

## Figures and Tables

**Table 1:** Results LCI [23].

Technological Operations	
1. Material feed	
Mass of the one preform PLA	18.24 g
Total energy consumption by three motors of the carousel	0.368 kWh
2. Heating the preform	
Energy of infrared lamp (100 kW)	3.2 kWh
Energy of infrared lamps (200 kW)	6.4 kWh
Energy of supply chain	0.16 kWh
3. Stretching and extension of the preform	
Amount of electrical energy consumed	6.95 kWh
Amount of compressed air needed to blow the preform	0.0016 kg/m^3^
4. Pressure shaping of the preforms	
Amount of electrical energy consumed	5.66 kWh
5. Degasification of the bottle	
Amount of electrical energy consumed	1.01 kWh
6. Bottle cooling	
Amount of electrical energy consumed	0.71 kWh
Water volume in a closed circulation	2.4 m^3^

**Table 2:** Results of characterizing environmental impacts of a bottle shaping process.

Impact Category	Unit	(CB)	(DB)	(PPS)	(PSE)	(PH)	(PBH)
Global warming, human health	DALY	4.5 × 10^−10^	1.2 × 10^−8^	8.9 × 10^−9^	7.2 × 10^−9^	1.2 × 10^−9^	2.1 × 10^−9^
Global warming. land ecosystems	species.yr	1.4 × 10^−12^	3.5 × 10^−11^	2.7 × 10^−11^	2.2 × 10^−11^	3.7 × 10^−12^	6.3 × 10^−12^
Global warming. fresh aquatic systems	species.yr	3.8 × 10^−17^	9.5 × 10^−16^	7.4 × 10^−16^	5.9 × 10^−16^	1.0 × 10^−16^	1.7 × 10^−16^
Startospheric ozone layer depletion	DALY	4.9 × 10^−14^	1.3 × 10^−12^	9.7 × 10^−13^	7.9 × 10^−13^	1.3 × 10^−13^	3.8 × 10^−13^
Ionizing radiation	DALY	6.1 × 10^−15^	1.6 × 10^−13^	1.2 × 10^−13^	1.0 × 10^−13^	1.6 × 10^−14^	2.1 × 10^−13^
Ozone formation. human health	DALY	7.4 × 10^−13^	1.9 × 10^−11^	1.4 × 10^−11^	1.2 × 10^−11^	2.0 × 10^−12^	3.9 × 10^−12^
Formation of fine particles	DALY	5.4 × 10^−10^	1.2 × 10^−8^	9.0 × 10^−9^	7.4 × 10^−9^	1.2 × 10^−9^	2.2 × 10^−9^
Formation of ozone land ecosystems	species.yr	1.1 × 10^−13^	2.6 × 10^−12^	2.1 × 10^−12^	1.6 × 10^−12^	2.8 × 10^−13^	5.6 × 10^−13^
Land acidification	species.yr	5.1 × 10^−13^	1.3 × 10^−11^	1.0 × 10^−11^	8.1 × 10^−12^	1.4 × 10^−12^	2.1 × 10^−12^
Eutrophication of fresh water	species.yr	3.5 × 10^−14^	9.1 × 10^−13^	6.9 × 10^−13^	5.7 × 10^−13^	9.5 × 10^−14^	1.3 × 10^−13^
Land ecotoxicity	species.yr	5.3 × 10^−15^	1.4 × 10^−13^	1.0 × 10^−13^	8.4 × 10^−14^	1.4 × 10^−14^	4.8 × 10^−14^
Fresh water ecotoxicity	species.yr	3.6 × 10^−17^	9.0 × 10^−16^	7.1 × 10^−16^	5.6 × 10^−16^	9.7 × 10^−17^	1.0 × 10^−15^
Marine water ecotoxicity	species.yr	2.8 × 10^−17^	7.1 × 10^−16^	5.5 × 10^−16^	4.4 × 10^−16^	7.5 × 10^−17^	4.4 × 10^−16^
Carcinogenic ecotoxicity for people	DALY	3.1 × 10^−12^	7.3 × 10^−11^	6.0 × 10^−11^	4.5 × 10^−11^	8.2 × 10^−12^	2.6 × 10^−10^
Non carcinogenic toxicity for people	DALY	3.6 × 10^−12^	9.2 × 10^−11^	7.0 × 10^−11^	5.7 × 10^−11^	9.6 × 10^−12^	3.3 × 10^−11^
Land use	species.yr	2.2 × 10^−13^	5.8 × 10^−12^	4.4 × 10^−12^	3.6 × 10^−12^	6.0 × 10^−13^	8.9 × 10^−13^
Deficiency of mineral resources	USD2013	5.1 × 10^−08^	1.0 × 10^−06^	8.5 × 10^−07^	6.4 × 10^−07^	1.2 × 10^−07^	2.5 × 10^−06^
Deficiency of fossil resources	USD2013	1.0 × 10^−05^	1.4 × 10^−04^	1.7 × 10^−04^	1.0 × 10^−04^	2.4 × 10^−05^	1.7 × 10^−05^
Use of water resources. human health	DALY	3.4 × 10^−11^	8.7 × 10^−10^	6.6 × 10^−10^	5.4 × 10^−10^	9.1 × 10^−11^	6.8 × 10^−09^
Use of water resources. land ecosystem	species.yr	2.1 × 10^−13^	5.3 × 10^−12^	4.0 × 10^−12^	3.3 × 10^−12^	5.5 × 10^−13^	4.2 × 10^−11^
Use of water resources. ecosystems	species.yr	9.2 × 10^−18^	2.4 × 10^−16^	1.8 × 10^−16^	1.5 × 10^−16^	2.5 × 10^−17^	1.9 × 10^−15^
	Emission level with the highest potential environmental impact						
	Emission level with higher potential environmental impact						
	Emission level with high potential environmental impact						
	Emission level with low potential environmental impact						
	Emission level with lower potential environmental impact						
	Emission level with the potential lowest environmental impact						

**Table 3:** Emission of ionizing radiation compounds into the air throughout the shaping cycle of PLA beverage bottles which is biodegradable in an industrial composting facility.

Substance	(CB)	(DB)	(PPS)	(PSE)	(PH)	(PBH)
Radioactive compounds	6.55 × 10^−14^	2.69 × 10^−16^	1.62 × 10^−16^	1.66 × 10^−16^	2.20 × 10^−17^	2.95 × 10^−16^
Carbon-14	3.50 × 10^−12^	9.58 × 10^−14^	7.17 × 10^−14^	5.93 × 10^−14^	9.78 × 10^−15^	1.18 × 10^−13^
Cesium-134	2.62 × 10^−19^	9.24 × 10^−21^	6.88 × 10^−21^	5.72 × 10^−21^	9.40 × 10^−22^	9.23 × 10^−21^
Cesium-137	9.91 × 10^−15^	1.08 × 10^−16^	7.70 × 10^−17^	6.65 × 10^−17^	1.05 × 10^−17^	2.68 × 10^−16^
Cobalt-58	2.59 × 10^−20^	6.61 × 10^−22^	4.89 × 10^−22^	4.09 × 10^−22^	6.68 × 10^−32^	8.89 × 10^−22^
Cobalt-60	7.57 × 10^−18^	2.21 × 10^−19^	1.64 × 10^−19^	1.37 × 10^−19^	2.24 × 10^−20^	2.62 × 10^−19^
Tritium	6.65 × 10^−15^	2.09 × 10^−16^	1.58 × 10^−16^	1.30 × 10^−16^	2.15 × 10^−17^	3.96 × 10^−16^
Iodine-129	1.02 × 10^−14^	2.37 × 10^−16^	1.75 × 10^−16^	1.47 × 10^−16^	2.39 × 10^−17^	4.12 × 10^−16^
Iodine-131	2.29 × 10^−16^	5.34 × 10^−17^	4.04 × 10^−17^	3.31 × 10^−17^	5.52 × 10^−18^	3.10 × 10^−17^
Iodine-133	4.22 × 10^−20^	8.67 × 10^−22^	6.38 × 10^−22^	5.37 × 10^−22^	8.71 × 10^23^	1.43 × 10^−21^
Krypton-85	3.02 × 10^−18^	5.98 × 10^−19^	4.53 × 10^−19^	3.71 × 10^−19^	6.18 × 10^−20^	3.55 × 10^−19^
Noble radioactive gases	7.39 × 10^−14^	1.71 × 10^−15^	1.26 × 10^−15^	1.06 × 10^−15^	1.73 × 10^−16^	2.98 × 10^−15^
Polonium-210	2.94 × 10^−14^	3.01 × 10^−15^	2.26 × 10^−15^	1.86 × 10^−15^	3.09 × 10^−16^	4.86 × 10^−16^
Radon-222	1.45 × 10^−12^	4.81 × 10^−14^	3.61 × 10^−14^	2.98 × 10^−14^	4.92 × 10^−15^	7.71 × 10^−14^
Xenon-133	2.75 × 10^−16^	7.26 × 10^−18^	5.38 × 10^−18^	4.50 × 10^−18^	7.35 × 10^−19^	9.73 × 10^−18^

**Table 4:** Emission of ionizing radiation compounds to water throughout the entire shaping cycle of PLA beverage bottles is biodegradable in an industrial composting facility.

Substance	(CB)	(DB)	(PPS)	(PSE)	(PH)	(PBH)
Iodine-131	8.21 × 10^−16^	2.84 × 10^−18^	1.69 × 10^−18^	1.75 × 10^−18^	2.31 × 10^−19^	9.20 × 10^−18^
Radioactive compounds	6.93 × 10^−19^	1.60 × 10^−20^	1.19 × 10^−20^	9.92 × 10^−21^	1.62 × 10^−21^	2.79 × 10^−20^
Antimony-124	6.88 × 10^−15^	2.31 × 10^−17^	1.37 × 10^−17^	1.42 × 10^−17^	1.86 × 10^−18^	7.68 × 10^−17^
Carbon-14	9.41 × 10^−17^	2.92 × 10^−19^	1.69 × 10^−19^	1.80 × 10^−19^	2.30 × 10^−20^	1.01 × 10^−18^
Tritium	3.52 × 10^−14^	2.57 × 10^−16^	1.77 × 10^−16^	1.59 × 10^−16^	2.41 × 10^−17^	6.29 × 10^−16^
Manganese-54	3.47 × 10^−17^	1.06 × 10^−18^	7.90 × 10^−19^	6.58 × 10^−19^	1.08 × 10^−19^	1.28 × 10^−18^
Strontium-90	1.60 × 10^−14^	3.88 × 10^−15^	2.94 × 10^−15^	2.41 × 10^−15^	4.02 × 10^−16^	2.24 × 10^−15^

**Table 5:** Results of characterization of environmental impacts for the category of global warming, (DALY).

Substance	(CB)	(DB)	(PPS)	(PSE)	(PH)	(PBH)
CO_2_	7.68 × 10^−8^	1.05 × 10^−8^	8.14 × 10^−9^	6.54 × 10^−9^	1.11 × 10^−9^	1.88 × 10^−9^
Chloroform	3.72 × 10^−14^	1.57 × 10^−16^	1.23 × 10^−16^	9.78 × 10^−17^	1.68 × 10^−17^	3.36 × 10^−16^
Nitric oxide	7.48 × 10^−09^	5.57 × 10^−11^	4.2 × 10^−11^	3.45 × 10^−11^	5.74 × 10^−12^	1.5 × 10^−11^
Chlorinated hydrocarbons	3.72 × 10^−14^	1.57 × 10^−16^	1.23 × 10^−16^	9.78 × 10^−17^	1.68 × 10^−17^	3.36 × 10^−16^
Methane	7 × 10^−15^	4.07 × 10^−17^	3.61 × 10^−17^	2.53 × 10^−17^	4.92 × 10^−18^	5.33 × 10^−17^
Nitrogen fluoride	9.1 × 10^−17^	9.28 × 10^−19^	2.53 × 10^−19^	5.64 × 10^−19^	3.43 × 10^−20^	5.01 × 10^−19^
Sulfur hexafluoride	2.65 × 10^−10^	3.56 × 10^−11^	2.67 × 10^−11^	2.2 × 10^−11^	3.65 × 10^−12^	7.3 × 10^−12^

**Table 6:** Results of characterization of environmental impacts on category of particulate matter formation (DALY).

Substance	(CB)	(DB)	(PPS)	(PSE)	(PH)	(PBH)
Particulate matter formation < 2.5um	7.13 × 10^−8^	2.1 × 10^−9^	1.56 × 10^−9^	1.3 × 10^−9^	2.14 × 10^−10^	6.13 × 10^−10^
Sulfur dioxide	5.75 × 10^−8^	9.76 × 10^−9^	7.46 × 10^−9^	6.05 × 10^−9^	1.02 × 10^−9^	1.54 × 10^−9^
Sulfur oxides	3.08 × 10^−13^	3.68 × 10^−15^	2.67 × 10^−15^	2.28 × 10^−15^	3.64 × 10^−16^	5.78 × 10^−15^
Sulfur trioxide	2.1 × 10^−13^	5.15 × 10^−17^	3.9 × 10^−17^	3.19 × 10^−17^	5.32 × 10^−18^	3.27 × 10^−17^

**Table 7:** Results of environmental impact characterization for the category of use of water resources (DALY).

Substance	(CB)	(DB)	(PPS)	(PSE)	(PH)	(PBH)
Water PL	−1.9 × 10^−9^	−2.3 × 10^−8^	−1.7 × 10^−8^	−1.4 × 10^−8^	−2.4 × 10^−9^	−2.1 × 10^−9^
Water in lakes PL	2.98 × 10^−19^	3.16 × 10^−18^	2.39 × 10^−18^	1.96 × 10^−18^	3.26 × 10^−19^	2.88 × 10^−19^
Water in rivers PL	6.85 × 10^−16^	7.26 × 10^−15^	5.49 × 10^−15^	4.5 × 10^−15^	7.5 × 10^−16^	6.62 × 10^−16^

**Table 8:** Results of environmental impacts characterization for the category of global warming, land ecosystems (species.yr).

Substance	(CB)	(DB)	(PPS)	(PSE)	(PH)	(PBH)
CO_2_	2.3162 × 10^−10^	3.1827 × 10^−11^	2.455 × 10^−11^	1.9735 × 10^−11^	3.3525 × 10^−12^	5.6821 × 10^−12^
Chloroform	1.1198 × 10^−16^	4.7415 × 10^−19^	3.7126 × 10^−19^	2.9447 × 10^−19^	5.0587 × 10^−20^	1.0102 × 10^−18^
Nitric oxide	2.2508 × 10^−11^	1.6776 × 10^−13^	1.266 × 10^−13^	1.0395 × 10^−13^	1.7281 × 10^−14^	4.5046 × 10^−14^
Chlorinated Hydrocarbons	5.5791 × 10^−16^	9.1828 × 10^−18^	7.561 × 10^−18^	5.7239 × 10^−18^	1.0271 × 10^−18^	7.9708 × 10^−18^
Methane	2.1078 × 10^−17^	1.2247 × 10^−19^	1.0875 × 10^−19^	7.6347 × 10^−20^	1.4835 × 10^−20^	1.6051 × 10^−19^
Nitrogen fluoride	2.7452 × 10^−19^	2.8002 × 10^−22^	7.6257 × 10^−22^	1.702 × 10^−21^	1.0352 × 10^−22^	1.5108 × 10^−21^
Sulfur Hexofluoride	7.9944 × 10^−13^	1.073 × 10^−13^	8.0621 × 10^−14^	6.6478 × 10^−14^	1.1006 × 10^−14^	2.2029 × 10^−14^

**Table 9:** Results of characterization of environmental impacts for the land acidification category (species.yr).

Substance	(CB)	(DB)	(PPS)	(PSE)	(PH)	(PBH)
Ammonia	2.88 × 10^−11^	1.18 × 10^−13^	8.96 × 10^−14^	7.33 × 10^−14^	1.22 × 10^−14^	2.63 × 10^−14^
Nitrate	1.65 × 10^−16^	1.43 × 10^−18^	1.2 × 10^−18^	8.92 × 10^−19^	1.64 × 10^−19^	2.18 × 10^−18^
Nitric oxides	1.6 × 10^−11^	1.56 × 10^−12^	1.2 × 10^−12^	9.65 × 10^−13^	1.64 × 10^−13^	3.2 × 10^−13^
Sulfur dioxide	6.7 × 10^−11^	1.14 × 10^−11^	8.69 × 10^−12^	7.05 × 10^−12^	1.19 × 10^−12^	1.79 × 10^−12^
Sulfur oxides	3.59 × 10^−16^	4.28 × 10^−18^	3.11 × 10^−18^	2.65 × 10^−18^	4.24 × 10^−19^	6.73 × 10^−18^
Sulfur trioxide	2.46 × 10^−16^	6.04 × 10^−20^	4.57 × 10^−20^	3.74 × 10^−20^	6.23 × 10^−21^	3.83 × 10^−20^
Sulfuric acid	2.88 × 10^−11^	1.18 × 10^−13^	8.96 × 10^−14^	7.33 × 10^−13^	1.22 × 10^−14^	2.63 × 10^−14^

**Table 10:** Results of environmental impact characterizing for the category of land use (species.yr).

Substance	(CB)	(DB)	(PPS)	(PSE)	(PH)	(PBH)
Annual crops	2.64 × 10^−10^	6.94 × 10^−15^	5.26 × 10^−15^	4.3 × 10^−15^	7.18 × 10^−16^	2.13 × 10^−15^
Forest	5.93 × 10^−14^	1.77 × 10^−15^	1.18 × 10^−15^	1.09 × 10^−15^	1.61 × 10^−16^	1.85 × 10^−15^
Industrial areas	8.82 × 10^−12^	5.71 × 10^−14^	4.37 × 10^−14^	3.54 × 10^−14^	5.97 × 10^−15^	3.61 × 10^−14^
Places of mineral extraction	5.14 × 10^−16^	2.94 × 10^−18^	2.56 × 10^−18^	1.83 × 10^−18^	3.5 × 10^−19^	1.41 × 10^−18^
Graze lands	1.89 × 10^−16^	2.59 × 10^−18^	2.12 × 10^−18^	1.62 × 10^−18^	2.87 × 10^−19^	1.57 × 10^−18^
Forest transformations	7.94 × 10^−13^	1.27 × 10^−14^	9.61 × 10^−15^	7.89 × 10^−15^	1.31 × 10^−15^	3.47 × 10^−15^
Green land Transformations (unused)	2.35 × 10^−15^	1.98 × 10^−17^	1.5 × 10^−17^	1.23 × 10^−17^	2.04 × 10^−18^	1.12 × 10^−17^

**Table 11:** Results of characterization of environmental impacts for the category of fossil fuel deficiency (USD2013).

Substance	(CB)	(DB)	(PPS)	(PSE)	(PH)	(PBH)
Coal	0.000647	0.000143	0.000108	8.87 × 10^−5^	1.48 × 10^−5^	2.16 × 10^−5^
Oil	0.001924	−2.5 × 10^−5^	2.9 × 10^−5^	−1.4 × 10^−5^	3.95 × 10^−6^	2.47 × 10^−5^
Natural gas	0.0041	1.72 × 10^−5^	3.6 × 10^−5^	1.13 × 10^−5^	4.92 × 10^−6^	3.12 × 10^−5^

## Supplementary Materials

The following are available online at https://www.mdpi.com/2073-4360/12/2/388/s1. Figure S1: Results of the characterization of environmental consequences for the category of scarcity of fossil resources obtained as a result of the PLA bottle shaping process (own study). Figure S2: Characterization results of environmental consequences for the category of ionizing radiation obtained as a result of the PLA bottle shaping process (own study). Figure S3: The results of the characterization of the environmental consequences for the carcinogenic toxicity category in humans obtained as a result of the PLA bottle shaping process (own study). Figure S4: Characterization results of environmental consequences for the category of non-creative toxicity to humans obtained as a result of the PLA bottle shaping process (own study). Figure S5: Characterization results of environmental consequences for the category of marine ecotoxicity obtained as a result of the PLA bottle shaping process (own study). Figure S6: The results of the characterization of the environmental consequences for the terrestrial ecotoxicity category obtained as a result of the PLA bottle shaping process (own study). Figure S7: The results of the characterization of environmental consequences for the category of mineral resource deficiency obtained as a result of the PLA bottle shaping process (own study). Figure S8: Results of the characterization of environmental consequences for the category of ozone formation affecting drow people obtained as a result of the process of shaping PLA bottles (own study). Figure S9: Results of characterization of environmental consequences for the ozone formation category affecting the ecosystem obtained as a result of the PLA bottle shaping process (own study). Figure S10: Results of the characterization of environmental consequences for the ozone layer depletion category obtained as a result of the PLA bottle shaping process (own study). Figure S11: Results of the characterization of the environmental consequences for the category of fresh water eutrophication obtained as a result of the PLA bottle shaping process (own study). Figure S12: Results of the characterization of the environmental consequences for the freshwater ecotoxicity category obtained as a result of the PLA bottle shaping process (own study). Table S1: Results of the characterization of environmental consequences for the category of scarcity of fossil resources obtained as a result of the PLA bottle shaping process (own study). Table S2: Characterization results of environmental consequences for the category of ionizing radiation obtained as a result of the PLA bottle shaping process (own study). Table S3: The results of the characterization of the environmental consequences for the carcinogenic toxicity category in humans obtained as a result of the PLA bottle shaping process (own study). Table S4: Characterization results of environmental consequences for the category of non-creative toxicity to humans obtained as a result of the PLA bottle shaping process (own study). Table S5: Characterization results of environmental consequences for the category of marine ecotoxicity obtained as a result of the PLA bottle shaping process (own study). Table S6: The results of the characterization of the environmental consequences for the terrestrial ecotoxicity category obtained as a result of the PLA bottle shaping process (own study). Table S7: The results of the characterization of environmental consequences for the category of mineral resource deficiency obtained as a result of the PLA bottle shaping process (own study). Table S8: Results of the characterization of environmental consequences for the category of ozone formation affecting drow people obtained as a result of the process of shaping PLA bottles (own study). Table S9: Results of characterization of environmental consequences for the ozone formation category affecting the ecosystem obtained as a result of the PLA bottle shaping process (own study). Table S10: Results of the characterization of environmental consequences for the ozone layer depletion category obtained as a result of the PLA bottle shaping process (own study). Table S11: Results of the characterization of the environmental consequences for the category of fresh water eutrophication obtained as a result of the PLA bottle shaping process (own study). Table S12: Results of the characterization of the environmental consequences for the freshwater ecotoxicity category obtained as a result of the PLA bottle shaping process (own study).
