# Supplementary material for: Application of LCA Method for Assessment of Environmental Impacts of a Polylactide (PLA) Bottle Shaping
Source: Polymers (Basel). 2020 Feb 9;12(2):388. doi: 10.3390/polym12020388 (PMC7077684; doi:10.3390/polym12020388)
Supplement: Supplementary file 1 [file polymers-12-00388-s001.pdf]

polymers

MDPI

Supplementary

Application of LCA Method for Assessment of Environmental Impacts of a Polylactide (PLA) Bottle Shaping

Patrycja Bałdowska-Witos [1,*], Weronika Kruszelnicka [1], Robert Kasner [1], Andrzej Tomporowski[1], Józef Flizikowski [1], Zbigniew Kłos [2], Katarzyna Piotrowska [3] and Katarzyna Markowska [4]

[1] Department of Technical Systems Engineering, Faculty of Mechanical Engineering, University of Science and Technology in Bydgoszcz, 85-796 Bydgoszcz, Poland; weronika.kruszelnicka@utp.edu.pl (W.K.), robert.kasner@gmail.com (R.K.), a.tomporowski@utp.edu.pl (A.T.), fliz@utp.edu.pl (J.F.)
[2] Institute of Machines and Motor Vehicles, Faculty of Transport Engineering, Poznan University of Technology, 60-965 Poznan, Poland; zbigniew.klos@put.poznan.pl
[3] Faculty of Mechanical Engineering, Lublin University of Technology; 20-618 Lublin, Poland; k.piotrowska@pollub.pl
[4] Department of Logistics and Transport Technologies, Faculty of Transport and Aviation Engineering, Silesian University of Technology, 40-019 Katowice, Poland; Katarzyna.Markowska@polsl.pl
* Correspondence: patrycja.baldowska-witos@utp.edu.pl

Received: 19 December 2019; Accepted: 2 February 2020; Published:

Table S1. Results of the characterization of environmental consequences for the category of scarcity of fossil resources obtained as a result of the PLA bottle shaping process (own study).

Substance	Unit	(CB)	(DB)	(PPS)	(PSE)	(PH)	(PBH)
carbon	USD2013	6.47×10^{-4}	1.43×10^{-4}	1.08×10^{-4}	8.87×10^{-5}	1.48×10^{-5}	2.16×10^{-5}
petroleum	USD2013	1.92×10^{-4}	-2.5×10^{-5}	2.9×10^{-5}	$--1.4 \times 10^{-05}$	3.95×10^{-5}	2.47×10^{-5}
natural gas	USD2013	4.10×10^{-3}	1.72×10^{-5}	3.6×10^{-5}	1.13×10^{-5}	4.92×10^{-6}	3.12×10^{-5}

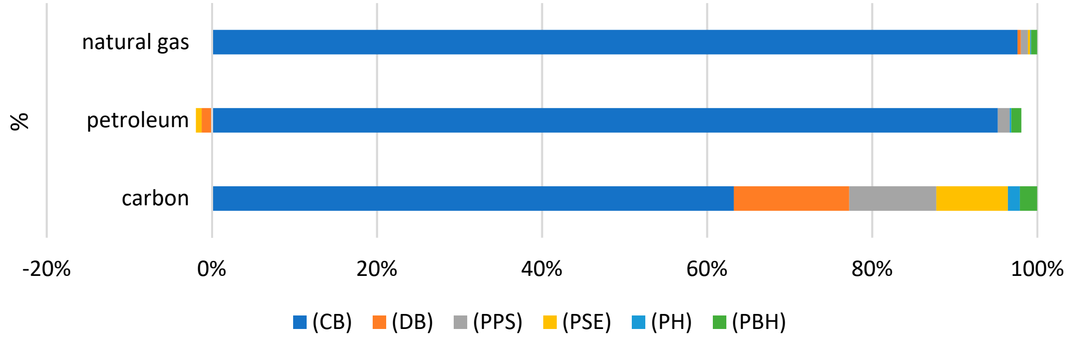

Figure S1. Results of the characterization of environmental consequences for the category of scarcity of fossil resources obtained as a result of the PLA bottle shaping process (own study).

Table S2. Characterization results of environmental consequences for the category of ionizing radiation obtained as a result of the PLA bottle shaping process (own study).

Substance	Unit	(CB)	(DB)	(PPS)	(PSE)	(PH)	(PBH)
radioactive compounds	DALY	6.55×10^{-14}	2.69×10^{-16}	1.62×10^{-16}	1.66×10^{-16}	2.2×10^{-17}	2.95×10^{-16}
radioactive compounds	DALY	6.93×10^{-19}	1.6×10^{-20}	1.19×10^{-20}	9.92×10^{-21}	1.62×10^{-21}	2.79×10^{-20}
antimony-124	DALY	6.88×10^{-15}	2.31×10^{-17}	1.37×10^{-17}	1.42×10^{-17}	1.86×10^{-18}	7.68×10^{-17}
carbon-14	DALY	3.5×10^{-12}	9.58×10^{-14}	7.17×10^{-14}	5.93×10^{-14}	9.78×10^{-15}	1.18×10^{-13}
carbon-14	DALY	9.41×10^{-17}	2.92×10^{-19}	1.69×10^{-19}	1.8×10^{-19}	2.3×10^{-20}	1.01×10^{-18}
cesium-134	DALY	2.62×10^{-19}	9.24×10^{-21}	6.88×10^{-21}	5.72×10^{-21}	9.4×10^{-22}	9.23×10^{-21}
cesium-137	DALY	9.91×10^{-15}	1.08×10^{-16}	7.7×10^{-17}	6.65×10^{-17}	1.05×10^{-17}	2.68×10^{-16}
cobalt-58	DALY	2.59×10^{-20}	6.61×10^{-22}	4.89×10^{-22}	4.09×10^{-22}	6.68×10^{-23}	8.89×10^{-22}
cobalt-60	DALY	7.57×10^{-18}	2.21×10^{-19}	1.64×10^{-19}	1.37×10^{-19}	2.24×10^{-20}	2.62×10^{-19}
tritium	DALY	6.65×10^{-15}	2.09×10^{-16}	1.58×10^{-16}	1.3×10^{-16}	2.15×10^{-17}	3.96×10^{-16}
tritium	DALY	3.52×10^{-14}	2.57×10^{-16}	1.77×10^{-16}	1.59×10^{-16}	2.41×10^{-17}	6.29×10^{-16}
iodine-129	DALY	1.02×10^{-14}	2.37×10^{-16}	1.75×10^{-16}	1.47×10^{-16}	2.39×10^{-17}	4.12×10^{-16}
iodine-131	DALY	2.29×10^{-16}	5.34×10^{-17}	4.04×10^{-17}	3.31×10^{-17}	5.52×10^{-18}	3.1×10^{-17}
iodine-131	DALY	8.21×10^{-16}	2.84×10^{-18}	1.69×10^{-18}	1.75×10^{-18}	2.3×10^{-19}	9.2×10^{-18}
iodine-133	DALY	4.22×10^{-20}	8.67×10^{-22}	6.38×10^{-22}	5.37×10^{-22}	8.71×10^{-23}	1.43×10^{-21}
krypton-85	DALY	3.02×10^{-18}	5.98×10^{-19}	4.53×10^{-19}	3.71×10^{-19}	6.18×10^{-20}	3.55×10^{-19}
manganese-54	DALY	3.47×10^{-17}	1.06×10^{-18}	7.9×10^{-18}	6.58×10^{-19}	1.08×10^{-19}	1.28×10^{-18}
noble radioactive gases	DALY	7.39×10^{-14}	1.71×10^{-15}	1.26×10^{-15}	1.06×10^{-15}	1.73×10^{-16}	2.98×10^{-15}
polonium-210	DALY	2.94×10^{-14}	3.01×10^{-15}	2.26×10^{-15}	1.86×10^{-15}	3.09×10^{-16}	4.86×10^{-16}

radon-222	DALY	1.45 × 10⁻¹²	4.81 × 10⁻¹⁴	3.61 × 10⁻¹⁴	2.98 × 10⁻¹⁴	4.92 × 10⁻¹⁵	7.71 × 10⁻¹⁴
strontium-90	DALY	1.6 × 10⁻¹⁴	3.88 × 10⁻¹⁵	2.94 × 10⁻¹⁵	2.41 × 10⁻¹⁵	4.02 × 10⁻¹⁶	2.24 × 10⁻¹⁵
xenon-133	DALY	2.75 × 10⁻¹⁶	7.26 × 10⁻¹⁸	5.38 × 10⁻¹⁸	4.5 × 10⁻¹⁸	7.35 × 10⁻¹⁹	9.73 × 10⁻¹⁸

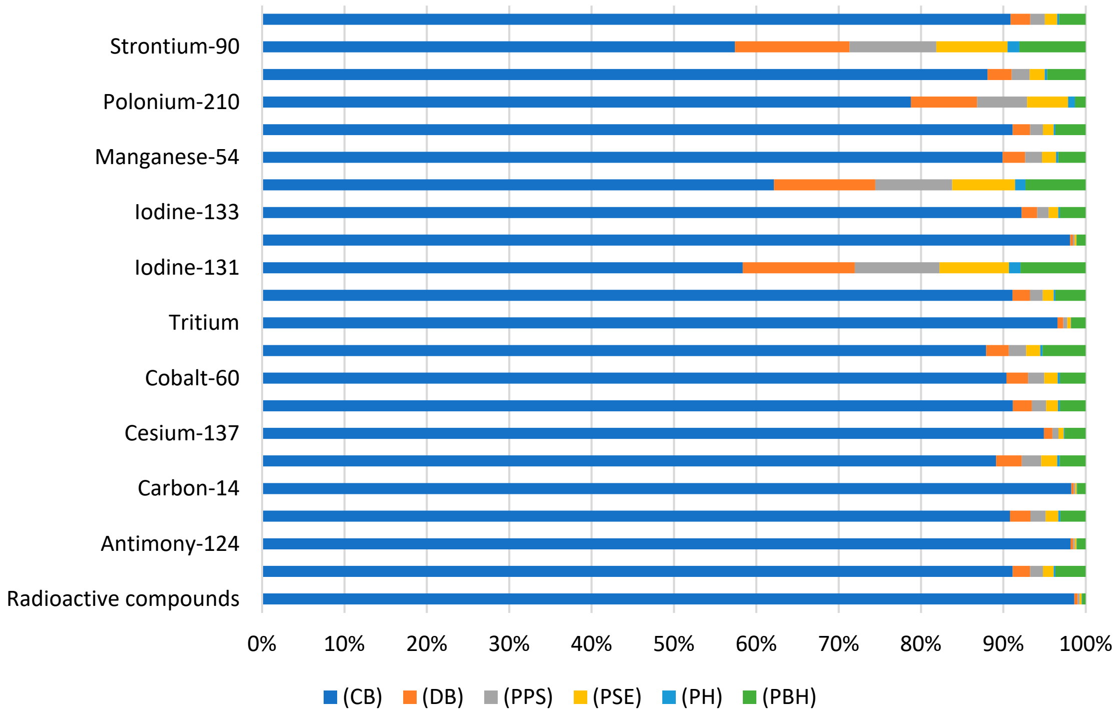

Figure S2. Characterization results of environmental consequences for the category of ionizing radiation obtained as a result of the PLA bottle shaping process (own study).

Table S3. Results of the characterization of environmental consequences for the carcinogenic toxicity category in humans obtained as a result of the PLA bottle shaping process (own study).

Substance	Unit	(CB)	(DB)	(PPS)	(PSE)	(PH)	(PBH)
acetaldehyde	DALY	4.76 × 10^{-14}	1.74 × 10^{-15}	1.38 × 10^{-15}	1.08 × 10^{-15}	1.88 × 10^{-16}	6.1 × 10^{-16}
aniline	DALY	1.63 × 10^{-17}	3.15 × 10^{-21}	2.37 × 10^{-21}	1.95 × 10^{-21}	3.24 × 10^{-22}	1.13 × 10^{-21}
benzene	DALY	1.11 × 10^{-15}	7.13 × 10^{-18}	6.18 × 10^{-18}	4.44 × 10^{-18}	8.43 × 10^{-19}	9.06 × 10^{-18}
cadmium	DALY	1.87 × 10^{-12}	5.53 × 10^{-14}	4.31 × 10^{-14}	3.43 × 10^{-14}	5.87 × 10^{-15}	4.68 × 10^{-14}
ethylene oxide	DALY	3.71 × 10^{-13}	-2.3 × 10^{-14}	5.63 × 10^{-15}	-1.4 × 10^{-14}	7.66 × 10^{-16}	4.81 × 10^{-15}
formaldehyde	DALY	2.14 × 10^{-11}	7.49 × 10^{-13}	5.73 × 10^{-13}	4.64 × 10^{-13}	7.83 × 10^{-14}	2.35 × 10^{-13}

furan	DALY	5.85 × 10^{-12}	3.71 × 10^{-15}	2.81 × 10^{-15}	2.3 × 10^{-15}	3.83 × 10^{-16}	1.43 × 10^{-15}
aromatic hydrocarbons	DALY	2.03 × 10^{-14}	2.01 × 10^{-14}	2 × 10^{-14}	1.97 × 10^{-14}	1.94 × 10^{-14}	1.89 × 10^{-14}
mercury	DALY	1.13 × 10^{-12}	1.03 × 10^{-12}	1.01 × 10^{-12}	8.8 × 10^{-13}	7.3 × 10^{-13}	5.29 × 10^{-13}
naphthalene	DALY	2.67 × 10^{-17}	2.61 × 10^{-17}	2.03 × 10^{-19}	1.54 × 10^{-19}	1.26 × 10^{-19}	2.1 × 10^{-20}
nickel	DALY	1.04 × 10^{-10}	4.6 × 10^{-12}	3.73 × 10^{-12}	2.86 × 10^{-12}	5.09 × 10^{-13}	1.38 × 10^{-12}

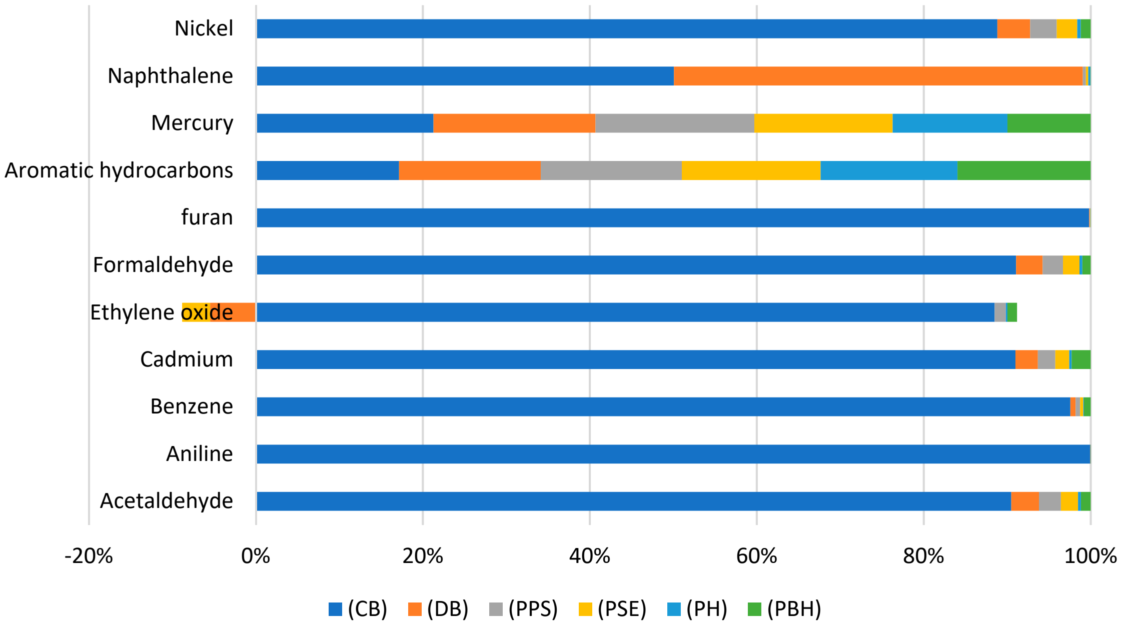

Figure S3. Results of the characterization of environmental consequences for the carcinogenic toxicity category in humans obtained as a result of the PLA bottle shaping process (own study).

Table S4. Results of the characterization of the environmental consequences for the category of non-creative toxicity to humans obtained as a result of the PLA bottle shaping process (own study).

Substance	Unit	(CB)	(DB)	(PPS)	(PSE)	(PH)	(PBH)
butyl alcohol	DALY	1.2 × 10^{-19}	1.4 × 10^{-23}	1.05 × 10^{-23}	8.68 × 10^{-24}	1.43 × 10^{-24}	8.77 × 10^{-24}
acetone	DALY	8.84 × 10^{-17}	1.79 × 10^{-18}	1.37 × 10^{-18}	1.11 × 10^{-18}	1.87 × 10^{-19}	1.05 × 10^{-18}
acetonitrile	DALY	1.55 × 10^{-16}	9.84 × 10^{-20}	7.45 × 10^{-20}	6.1 × 10^{-20}	1.02 × 10^{-20}	3.79 × 10^{-20}
acrylic acid	DALY	1.82 × 10^{-20}	-3 × 10^{-23}	1.85 × 10^{-23}	-1.7 × 10^{-23}	2.35 × 10^{-24}	5.2 × 10^{-23}
benzene	DALY	5.08 × 10^{-15}	5.49 × 10^{-16}	4.17 × 10^{-16}	3.4 × 10^{-16}	5.69 × 10^{-17}	2.45 × 10^{-16}

cadmium	DALY	7.42 × 10−11	2.44 × 10−12	1.9 × 10−12	1.52 × 10−12	2.59 × 10−13	9.39 × 10−13
carboxylic acid	DALY	2.39 × 10−15	4.97 × 10−17	3.8 × 10−17	3.08 × 10−17	5.19 × 10−18	3.11 × 10−17
copper	DALY	8.12 × 10−13	8.05 × 10−13	8.02 × 10−13	7.83 × 10−13	7.63 × 10−13	7.32 × 10−13
formaldehyde	DALY	6.23 × 10−14	3.68 × 10−15	2.79 × 10−15	2.28 × 10−15	3.8 × 10−16	6.23 × 10−16
furan	DALY	2.37 × 10−14	1.5 × 10−17	1.14 × 10−17	9.31 × 10−18	1.55 × 10−18	5.79 × 10−18
mercury	DALY	2.89 × 10−11	2.67 × 10−11	2.61 × 10−11	2.27 × 10−11	1.86 × 10−11	1.31 × 10−11
methanol	DALY	2.33 × 10−17	1.78 × 10−18	1.37 × 10−18	1.11 × 10−18	1.86 × 10−19	1.51 × 10−18
molybdenum	DALY	4.26 × 10−12	2.44 × 10−13	1.87 × 10−13	1.51 × 10−13	2.56 × 10−14	5.67 × 10−14
nickel	DALY	1.34 × 10−12	6.03 × 10−14	4.88 × 10−14	3.75 × 10−14	6.65 × 10−15	1.79 × 10−14
propylene oxide	DALY	9.87 × 10−19	5.78 × 10−21	4.38 × 10−21	3.58 × 10−21	5.97 × 10−22	2.97 × 10−21
silver	DALY	9.32 × 10−13	3.52 × 10−16	3.45 × 10−16	2.21 × 10−16	4.7 × 10−17	3.7 × 10−16
styrene	DALY	2.2 × 10−19	-5.8 × 10−20	1.12 × 10−21	-3.5 × 10−20	1.51 × 10−22	2.36 × 10−21
xylene	DALY	6.74 × 10−17	1.52 × 10−17	1.15 × 10−17	9.41 × 10−18	1.57 × 10−18	2.08 × 10−18

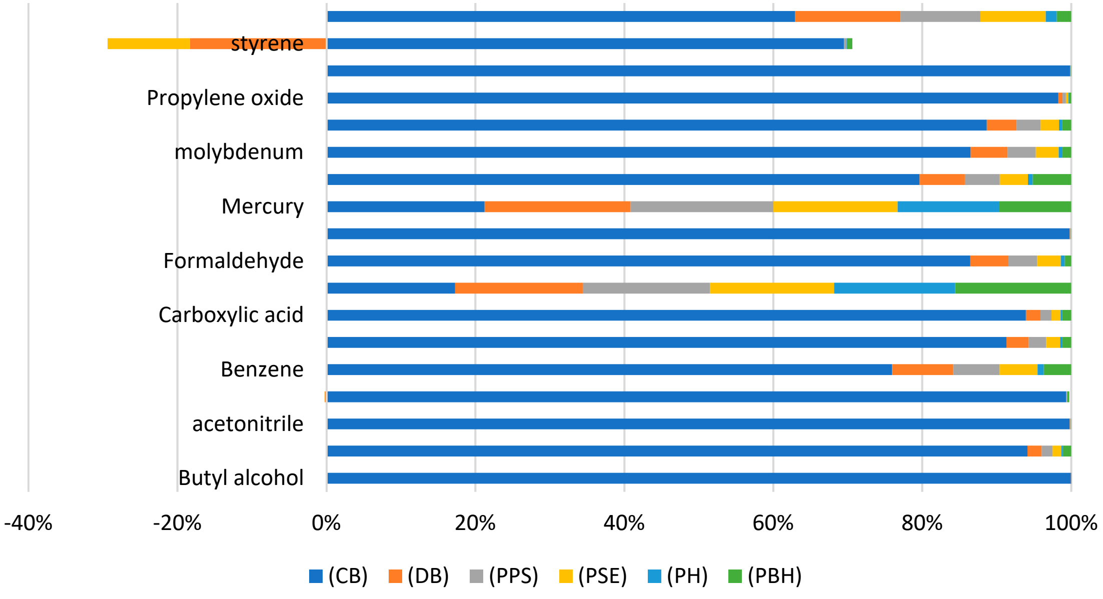

Figure S4. Results of the characterization of the environmental consequences for the category of non-creative toxicity to humans obtained as a result of the PLA bottle shaping process (own study).

Table S5. Characterization results of environmental consequences for the category of marine ecotoxicity obtained as a result of the PLA bottle shaping process (own study).

Substance	Unit	(CB)	(DB)	(PPS)	(PSE)	(PH)	(PBH)
butyl alcohol	species.yr	3.15×10^{-24}	3.15×10^{-24}	3.67×10^{-28}	2.74×10^{-28}	2.27×10^{-28}	3.74×10^{-29}
propyl alcohol	species.yr	3.58×10^{-23}	3.58×10^{-23}	1.07×10^{-27}	7.99×10^{-28}	6.62×10^{-28}	1.09×10^{-28}
acetaldehyde	species.yr	3.21×10^{-23}	3.21×10^{-23}	9.3×10^{-28}	6.9×10^{-28}	5.76×10^{-28}	9.41×10^{-29}
acetic acid	species.yr	8.43×10^{-23}	8.43×10^{-23}	2.44×10^{-27}	1.81×10^{-27}	1.51×10^{-27}	2.47×10^{-28}
acetic acid	species.yr	1.07×10^{-24}	1.01×10^{-24}	1.98×10^{-26}	1.5×10^{-26}	1.23×10^{-26}	2.05×10^{-27}
acetone	species.yr	7.69×10^{-21}	1.57×10^{-22}	1.2×10^{-22}	9.76×10^{-23}	1.64×10^{-23}	9.49×10^{-23}
acetonitrile	species.yr	5.4×10^{-24}	5.4×10^{-24}	5.4×10^{-24}	5.38×10^{-24}	5.38×10^{-24}	5.35×10^{-24}
acetyl chloride	species.yr	1.91×10^{-21}	1.91×10^{-21}	1.91×10^{-21}	1.9×10^{-21}	1.9×10^{-21}	1.89×10^{-21}
acrylic acid	species.yr	2.14×10^{-25}	2.13×10^{-25}	2.13×10^{-25}	2.12×10^{-25}	2.12×10^{-25}	2.11×10^{-25}
benzene	species.yr	2.02×10^{-19}	2.24×10^{-20}	1.7×10^{-20}	1.39×10^{-20}	2.33×10^{-21}	1.01×10^{-20}
cadmium	species.yr	3.18×10^{-19}	1.9×10^{-21}	1.44×10^{-21}	1.18×10^{-21}	1.97×10^{-22}	9.43×10^{-22}
carboxylic acid	species.yr	4.6×10^{-18}	7.45×10^{-20}	6.05×10^{-20}	4.64×10^{-20}	8.21×10^{-21}	3.81×10^{-20}
chloroacetic acid	species.yr	7.86×10^{-21}	8.66×10^{-25}	5.71×10^{-25}	5.35×10^{-25}	7.78×10^{-26}	5.74×10^{-25}
cobalt	species.yr	1.28×10^{-17}	5.06×10^{-19}	3.86×10^{-19}	3.14×10^{-19}	5.26×10^{-20}	1.71×10^{-19}
copper	species.yr	1.37×10^{-17}	3.26×10^{-19}	2.77×10^{-19}	2.03×10^{-19}	3.79×10^{-20}	6×10^{-20}
ethanol	species.yr	1.61×10^{-21}	3.78×10^{-23}	2.88×10^{-23}	2.34×10^{-23}	3.93×10^{-24}	9.88×10^{-23}
ethyl oxide	species.yr	2.52×10^{-23}	3.73×10^{-25}	3.27×10^{-25}	2.33×10^{-25}	4.47×10^{-26}	9.65×10^{-25}
formaldehyde	species.yr	7.16×10^{-19}	5.58×10^{-21}	4.22×10^{-21}	3.46×10^{-21}	5.76×10^{-22}	3.26×10^{-21}
formic acid	species.yr	1.81×10^{-23}	1.81×10^{-23}	1.81×10^{-23}	1.81×10^{-23}	1.81×10^{-23}	1.8×10^{-23}
mercury	species.yr	6.53×10^{-18}	6.48×10^{-18}	6.47×10^{-18}	6.38×10^{-18}	6.3×10^{-18}	6.16×10^{-18}

methanol	species.yr	4.67×10^{-22}	4.64×10^{-22}	4.63×10^{-22}	4.58×10^{-22}	4.54×10^{-22}	4.45×10^{-22}
methyl acetate	species.yr	5.66×10^{-19}	1.13×10^{-19}	8.69×10^{-20}	7.01×10^{-20}	1.19×10^{-20}	2.83×10^{-20}
molybdenum	species.yr	8.79×10^{-19}	8.51×10^{-19}	8.39×10^{-19}	7.65×10^{-19}	6.79×10^{-19}	5.61×10^{-19}
nickel	species.yr	9.62×10^{-16}	4.33×10^{-17}	3.5×10^{-17}	2.69×10^{-17}	4.78×10^{-18}	1.28×10^{-17}
propionic acid	species.yr	8.42×10^{-22}	1.13×10^{-23}	8.59×10^{-24}	7.01×10^{-24}	1.17×10^{-24}	3.05×10^{-24}
propylene oxide	species.yr	4.33×10^{-17}	1.12×10^{-17}	8.45×10^{-18}	6.92×10^{-18}	1.15×10^{-18}	1.41×10^{-18}
selenium	species.yr	3.18×10^{-18}	6.06×10^{-19}	4.72×10^{-19}	3.76×10^{-19}	6.44×10^{-20}	1.64×10^{-19}
silver	species.yr	1.09×10^{-15}	4.34×10^{-18}	3.11×10^{-18}	2.68×10^{-18}	4.24×10^{-19}	3.15×10^{-18}
sodium formate	species.yr	1.93×10^{-25}	1.88×10^{-25}	1.87×10^{-25}	1.86×10^{-25}	1.85×10^{-25}	1.82×10^{-25}
styrene	species.yr	2.24×10^{-23}	-5.4×10^{-24}	1.52×10^{-25}	-3.2×10^{-24}	2.06×10^{-26}	2.64×10^{-25}
sulphuric acid	species.yr	1.04×10^{-20}	2.86×10^{-23}	3.14×10^{-23}	1.81×10^{-23}	4.27×10^{-24}	3.98×10^{-22}
sulphuric acid	species.yr	2.63×10^{-22}	1.7×10^{-24}	1.31×10^{-24}	1.05×10^{-24}	1.79×10^{-25}	7.25×10^{-25}
xylene	species.yr	1.11×10^{-20}	2.68×10^{-21}	2.03×10^{-21}	1.66×10^{-21}	2.77×10^{-22}	3.66×10^{-22}
zinc	species.yr	5.19×10^{-15}	1.62×10^{-16}	1.26×10^{-16}	1.01×10^{-16}	1.72×10^{-17}	9.55×10^{-17}

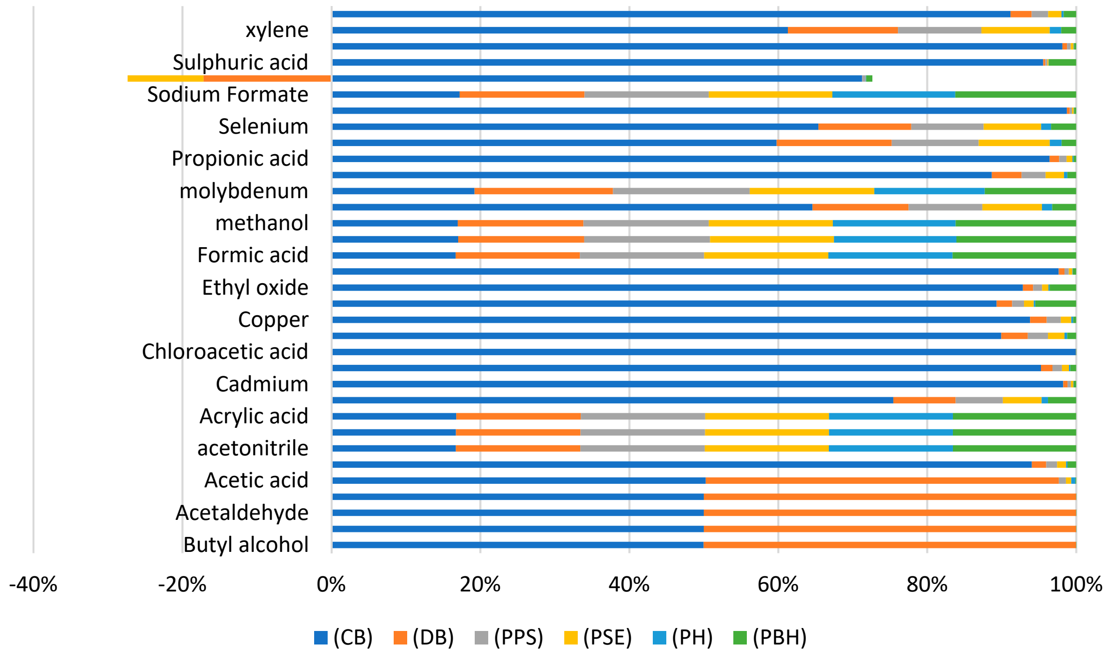

Figure S5. Characterization results of environmental consequences for the category of marine ecotoxicity obtained as a result of the PLA bottle shaping process (own study).

Table S6. The results of the characterization of the environmental consequences for the terrestrial ecotoxicity category obtained as a result of the PLA bottle shaping process (own study).

Substance	Unit	(CB)	(DB)	(PPS)	(PSE)	(PH)	(PBH)
butyl alcohol	species.yr	1.137×10^{-22}	1.358×10^{-26}	9.905×10^{-27}	8.205×10^{-27}	1.356×10^{-27}	8.286×10^{-27}
butyl alcohol	species.yr	1.637×10^{-22}	-4.11×10^{-23}	4.925×10^{-25}	-2.41×10^{-23}	6.798×10^{-26}	1.092×10^{-24}
amyl alcohol	species.yr	5.69×10^{-21}	5.69×10^{-21}	5.69×10^{-21}	5.68×10^{-21}	5.68×10^{-21}	5.65×10^{-21}
amyl alcohol	species.yr	3.5×10^{-21}	3.5×10^{-21}	3.5×10^{-21}	3.49×10^{-21}	3.49×10^{-21}	3.47×10^{-21}
propyl alcohol	species.yr	9.56×10^{-21}	9.42×10^{-21}	9.41×10^{-21}	9.33×10^{-21}	9.27×10^{-21}	9.14×10^{-21}
propyl alcohol	species.yr	2.59×10^{-22}	2.59×10^{-22}	2.59×10^{-22}	2.58×10^{-22}	2.58×10^{-22}	2.57×10^{-22}
isobutanol	species.yr	1.22×10^{-20}	1.22×10^{-20}	1.22×10^{-20}	1.21×10^{-20}	1.21×10^{-20}	1.21×10^{-20}
isobutanol	species.yr	9.1×10^{-21}	9.1×10^{-21}	9.1×10^{-21}	9.07×10^{-21}	9.07×10^{-21}	9.02×10^{-21}
mcpa	species.yr	1.97×10^{-21}	1.95×10^{-21}	1.95×10^{-21}	1.92×10^{-21}	1.89×10^{-21}	1.85×10^{-21}
mcpa	species.yr	8.06×10^{-29}	8×10^{-29}	7.99×10^{-29}	7.87×10^{-29}	7.76×10^{-29}	7.57×10^{-29}

mcpa	species.yr	2.14×10^{-25}	2.13×10^{-25}	2.13×10^{-25}	2.12×10^{-25}	2.11×10^{-25}	2.08×10^{-25}
acenaphthene	species.yr	4.09×10^{-21}	4.07×10^{-21}	4.06×10^{-21}	4.03×10^{-21}	4.01×10^{-21}	3.96×10^{-21}
acenaphthene	species.yr	8.25×10^{-22}	8.21×10^{-22}	8.2×10^{-22}	8.13×10^{-22}	8.08×10^{-22}	7.98×10^{-22}
acetic acid	species.yr	1.374×10^{-18}	2.759×10^{-20}	2.119×10^{-20}	1.702×10^{-20}	2.972×10^{-21}	9.192×10^{-21}
acetic acid	species.yr	7.024×10^{-20}	-2.54×10^{-21}	3.697×10^{-23}	-1.52×10^{-21}	4.913×10^{-24}	1.339×10^{-22}
acetone	species.yr	1.374×10^{-18}	2.759×10^{-20}	2.179×10^{-20}	1.782×10^{-20}	2.942×10^{-21}	9.492×10^{-21}
acetone	species.yr	7.024×10^{-20}	-2.50×10^{-21}	3.606×10^{-23}	-1.50×10^{-21}	4.993×10^{-24}	1.339×10^{-22}
acetonitrile	species.yr	3.688×10^{-19}	2.339×10^{-22}	1.7×10^{-22}	1.449×10^{-22}	2.41×10^{-23}	9.019×10^{-23}
acetonitrile	species.yr	2.423×10^{-22}	3.802×10^{-26}	2.846×10^{-26}	2.33×10^{-26}	3.878×10^{-27}	1.776×10^{-26}
acetyl chloride	species.yr	1.69×10^{-19}	1.69×10^{-19}	1.69×10^{-19}	1.69×10^{-19}	1.69×10^{-19}	1.68×10^{-19}
cadmium	species.yr	2.422×10^{-14}	7.851×10^{-16}	6.132×10^{-16}	4.879×10^{-16}	8.34×10^{-17}	3.483×10^{-16}
cadmium	species.yr	2.777×10^{-35}	5.769×10^{-37}	4.385×10^{-37}	3.547×10^{-37}	5.604×10^{-38}	7.137×10^{-37}
cadmium	species.yr	6.202×10^{-17}	4.306×10^{-19}	3.897×10^{-19}	2.753×10^{-19}	5.198×10^{-20}	5.537×10^{-19}
copper	species.yr	1.684×10^{-12}	5.788×10^{-14}	4.522×10^{-14}	3.594×10^{-14}	6.162×10^{-15}	1.692×10^{-14}
ethanol	species.yr	1.209×10^{-19}	3.272×10^{-21}	2.468×10^{-21}	2.028×10^{-21}	3.367×10^{-22}	4.843×10^{-21}
nickel	species.yr	2.195×10^{-13}	9.363×10^{-15}	7.666×10^{-15}	5.821×10^{-15}	1.045×10^{-15}	2.943×10^{-15}
selenium	species.yr	9.930×10^{-15}	2.461×10^{-15}	1.861×10^{-15}	1.525×10^{-15}	2.540×10^{-16}	3.132×10^{-16}
zinc	species.yr	1.848×10^{-13}	1.849×10^{-14}	1.411×10^{-14}	1.146×10^{-14}	1.926×10^{-15}	1.087×10^{-14}

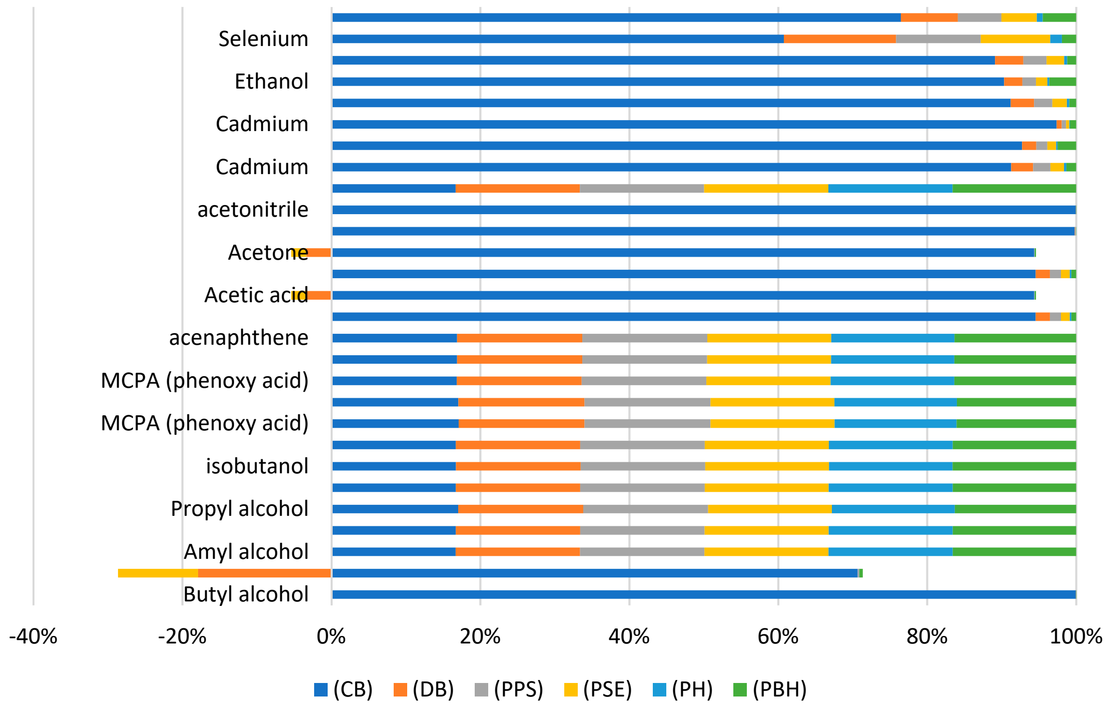

Figure S6. The results of the characterization of the environmental consequences for the terrestrial ecotoxicity category obtained as a result of the PLA bottle shaping process (own study).

Table S7. Results of the characterization of environmental consequences for the category of mineral resource deficiency obtained as a result of the PLA bottle shaping process (own study).

Substance	Unit	(CB)	(DB)	(PPS)	(PSE)	(PH)	(PBH)
aluminum	USD2013	1.6016×10^{-06}	3.1655×10^{-08}	2.6012×10^{-08}	1.9731×10^{-08}	3.5332×10^{-09}	2.4416×10^{-07}
baryta	USD2013	1.5027×10^{-07}	2.6218×10^{-09}	2.0012×10^{-09}	1.6252×10^{-09}	2.7316×10^{-10}	1.7696×10^{-09}
cadmium	USD2013	2.1194×10^{-08}	1.1197×10^{-10}	1.0156×10^{-10}	6.9945×10^{-11}	1.3829×10^{-11}	7.2944×10^{-10}
chrome	USD2013	5.0199×10^{-07}	5.3789×10^{-09}	4.663×10^{-09}	3.3582×10^{-09}	6.3378×10^{-10}	7.2244×10^{-09}
clay	USD2013	3.9667×10^{-08}	1.5118×10^{-09}	1.166×10^{-09}	9.3761×10^{-10}	1.5911×10^{-10}	4.5818×10^{-09}
cobalt	USD2013	2.1358×10^{-09}	5.155×10^{-11}	4.6315×10^{-11}	3.2157×10^{-11}	6.3158×10^{-12}	1.0863×10^{-10}
copper	USD2013	1.3267×10^{-06}	2.0922×10^{-08}	1.7054×10^{-08}	1.3046×10^{-08}	2.3137×10^{-09}	8.9667×10^{-09}
dolomite	USD2013	8.3569×10^{-12}	2.0261×10^{-13}	1.6522×10^{-13}	1.2603×10^{-13}	2.2508×10^{-14}	3.9457×10^{-13}
feldspar	USD2013	1.2212×10^{-12}	-3.2888×10^{-14}	1.6349×10^{-14}	-1.96×10^{-14}	2.2228×10^{-15}	1.0542×10^{-12}

gal	USD2013	6.0246×10^{-12}	5.4671×10^{-16}	6.9481×10^{-15}	5.1087×10^{-16}	9.4787×10^{-16}	1.8755×10^{-14}
gold	USD2013	2.6936×10^{-07}	-3.5082×10^{-10}	2.8025×10^{-10}	-1.943×10^{-10}	3.5615×10^{-11}	8.3833×10^{-10}
gypsum	USD2013	1.4293×10^{-08}	2.9327×10^{-10}	2.3987×10^{-10}	1.8222×10^{-10}	3.2739×10^{-11}	5.1951×10^{-10}
iodine	USD2013	3.1405×10^{-07}	2.1312×10^{-11}	1.5937×10^{-11}	1.3206×10^{-11}	2.1743×10^{-12}	8.8203×10^{-12}
manganese	USD2013	6.7424×10^{-08}	2.2928×10^{-09}	1.7851×10^{-09}	1.423×10^{-09}	2.4345×10^{-10}	2.1872×10^{-09}
molybdenum	USD2013	3.1695×10^{-07}	1.0963×10^{-08}	8.5573×10^{-09}	6.8042×10^{-09}	1.167×10^{-09}	6.1168×10^{-09}
nickel	USD2013	5.5634×10^{-07}	6.7287×10^{-09}	5.4613×10^{-09}	4.1942×10^{-09}	7.4121×10^{-10}	6.1615×10^{-09}
nacre	USD2013	1.4108×10^{-10}	-1.6318×10^{-12}	2.5157×10^{-12}	-9.112×10^{-13}	3.4267×10^{-13}	1.5687×10^{-11}
phosphorus	USD2013	3.2187×10^{-06}	6.3771×10^{-10}	5.1498×10^{-10}	3.9649×10^{-10}	7.0165×10^{-11}	2.0579×10^{-09}
platinum	USD2013	5.2672×10^{-08}	6.9492×10^{-10}	5.6682×10^{-10}	4.3331×10^{-10}	7.6911×10^{-11}	3.0388×10^{-10}
pumice	USD2013	5.5716×10^{-09}	1.3425×10^{-10}	1.0958×10^{-10}	8.3517×10^{-11}	1.4926×10^{-11}	2.5615×10^{-10}
silver	USD2013	2.4645×10^{-07}	1.302×10^{-09}	1.181×10^{-09}	8.1334×10^{-10}	1.6081×10^{-10}	8.4822×10^{-09}
strontium	USD2013	1.0924×10^{-10}	3.1237×10^{-13}	3.3658×10^{-13}	1.9696×10^{-13}	4.572×10^{-14}	4.0039×10^{-12}
talc	USD2013	5.1219×10^{-10}	1.2216×10^{-11}	9.6508×10^{-12}	7.5921×10^{-12}	1.3142×10^{-12}	4.5647×10^{-10}
zinc	USD2013	3.0525×10^{-07}	1.4586×10^{-09}	1.4625×10^{-09}	9.15×10^{-10}	1.9913×10^{-10}	1.05×10^{-08}

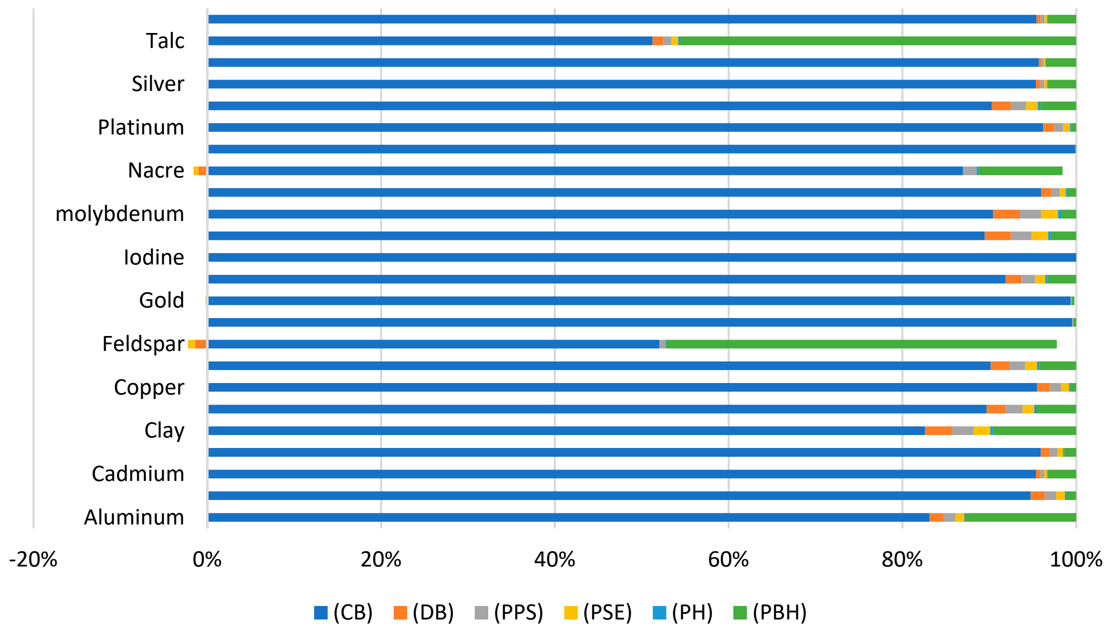

Figure S7. Results of the characterization of environmental consequences for the category of mineral resource deficiency obtained as a result of the PLA bottle shaping process (own study).

Table S8. Characterization results of environmental consequences for the category of ozone formation affecting drow people obtained as a result of the process of shaping PLA bottles (own study).

Substance	Unit	(CB)	(DB)	(PPS)	(PSE)	(PH)	(PBH)
butyl alcohol	DALY	8.85×10^{-18}	1.03×10^{-21}	7.71×10^{-22}	6.38×10^{-22}	1.05×10^{-22}	6.45×10^{-22}
propyl alcohol	DALY	2.71×10^{-17}	3.12×10^{-21}	2.54×10^{-21}	1.94×10^{-21}	3.46×10^{-22}	2.55×10^{-21}
acetaldehyde	DALY	2.28×10^{-14}	2.89×10^{-16}	2.27×10^{-16}	1.79×10^{-16}	3.1×10^{-17}	9.65×10^{-17}
acetic acid	DALY	1.27×10^{-14}	1.27×10^{-14}	1.26×10^{-14}	1.26×10^{-14}	1.25×10^{-14}	1.24×10^{-14}
acetone	DALY	2.41×10^{-15}	4.94×10^{-17}	3.78×10^{-17}	3.06×10^{-17}	5.15×10^{-18}	2.98×10^{-17}
butane	DALY	1.24×10^{-15}	2.27×10^{-17}	1.74×10^{-17}	1.41×10^{-17}	2.38×10^{-18}	1.43×10^{-17}
ethanol	DALY	4.35×10^{-15}	1.05×10^{-16}	8×10^{-17}	6.53×10^{-17}	1.09×10^{-17}	2.47×10^{-16}
formic acid	DALY	5.4×10^{-14}	3.05×10^{-15}	2.31×10^{-15}	1.89×10^{-15}	3.15×10^{-16}	5.45×10^{-16}
nitrogen oxides	DALY	1.9×10^{-10}	1.86×10^{-11}	1.44×10^{-11}	1.15×10^{-11}	1.96×10^{-12}	3.81×10^{-12}
propionic acid	DALY	3.13×10^{-16}	3.68×10^{-18}	2.77×10^{-18}	2.28×10^{-18}	3.78×10^{-19}	2.21×10^{-18}

toluene	DALY	8.57 × 10^{-14}	3.94 × 10^{-15}	2.98 × 10^{-15}	2.44 × 10^{-15}	4.07 × 10^{-16}	7.31 × 10^{-16}

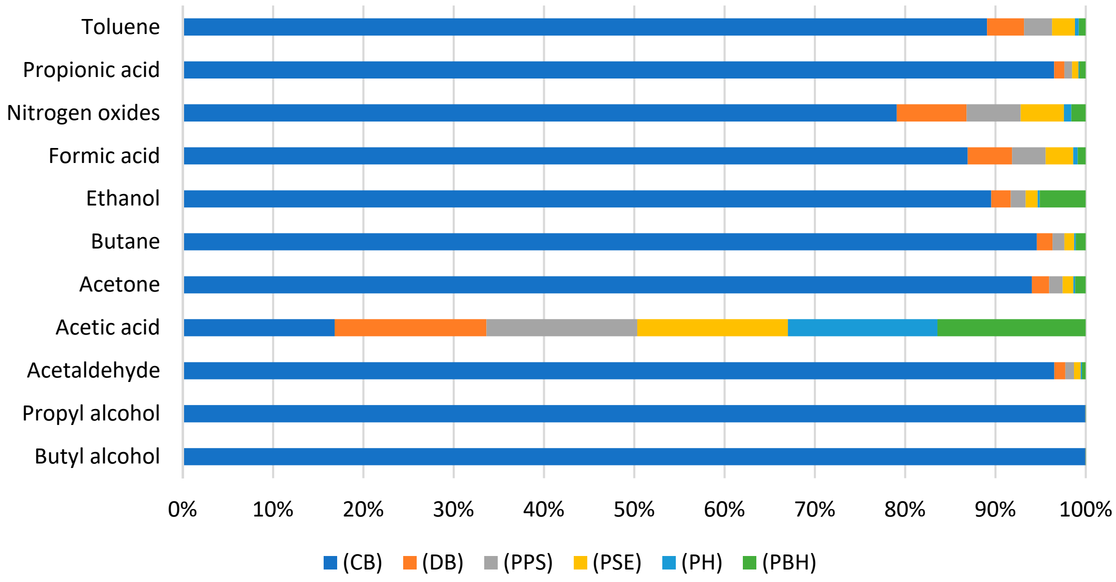

Figure S8. Characterization results of environmental consequences for the category of ozone formation affecting drow people obtained as a result of the process of shaping PLA bottles (own study).

Table S9. results of the characterization of the environmental consequences for the ozone formation category affecting the ecosystem obtained as a result of the PLA bottle shaping process (own study).

Substance	Unit	(CB)	(DB)	(PPS)	(PSE)	(PH)	(PBH)
butyl alcohol	DALY	2.02 × 10^{-18}	2.02 × 10^{-18}	2.35 × 10^{-22}	1.76 × 10^{-22}	1.45 × 10^{-22}	2.4 × 10^{-23}
propyl alcohol	DALY	6.19 × 10^{-18}	7.11 × 10^{-22}	5.78 × 10^{-22}	4.42 × 10^{-22}	7.89 × 10^{-23}	5.82 × 10^{-22}
acetaldehyde	DALY	5.4 × 10^{-15}	5.38 × 10^{-15}	5.37 × 10^{-15}	5.31 × 10^{-15}	5.26 × 10^{-15}	5.17 × 10^{-15}
acetic acid	DALY	2.90 × 10^{-15}	2.89 × 10^{-15}	2.89 × 10^{-15}	2.87 × 10^{-15}	2.86 × 10^{-15}	2.82 × 10^{-15}
acetone	DALY	5.51 × 10^{-16}	1.13 × 10^{-17}	8.62 × 10^{-18}	7 × 10^{-18}	1.18 × 10^{-18}	6.8 × 10^{-18}
butane	DALY	1.72 × 10^{-14}	3.06 × 10^{-16}	2.36 × 10^{-16}	1.9 × 10^{-16}	3.23 × 10^{-17}	1.65 × 10^{-16}
ethanol	DALY	9.95 × 10^{-16}	2.41 × 10^{-17}	1.83 × 10^{-17}	1.49 × 10^{-17}	2.5 × 10^{-18}	5.65 × 10^{-17}
formic acid	DALY	7.99 × 10^{-17}	1.32 × 10^{-18}	1.08 × 10^{-18}	8.2 × 10^{-19}	1.47 × 10^{-19}	1.14 × 10^{-18}
methanol	DALY	3.73 × 10^{-15}	-1.5 × 10^{-17}	1.08 × 10^{-17}	-8.4 × 10^{-18}	1.47 × 10^{-18}	7.08 × 10^{-18}

nitrogen oxides	DALY	2.76 × 10^{-16}	2.4 × 10^{-18}	2.01 × 10^{-18}	1.49 × 10^{-18}	2.74 × 10^{-19}	3.64 × 10^{-18}
propionic acid	DALY	-2.1 × 10^{-17}	-6 × 10^{-20}	-5.3 × 10^{-20}	-3.8 × 10^{-20}	-7.2 × 10^{-21}	-6.6 × 10^{-20}
toluene	DALY	1.96 × 10^{-14}	9.01 × 10^{-16}	6.82 × 10^{-16}	5.58 × 10^{-16}	9.31 × 10^{-17}	1.67 × 10^{-16}

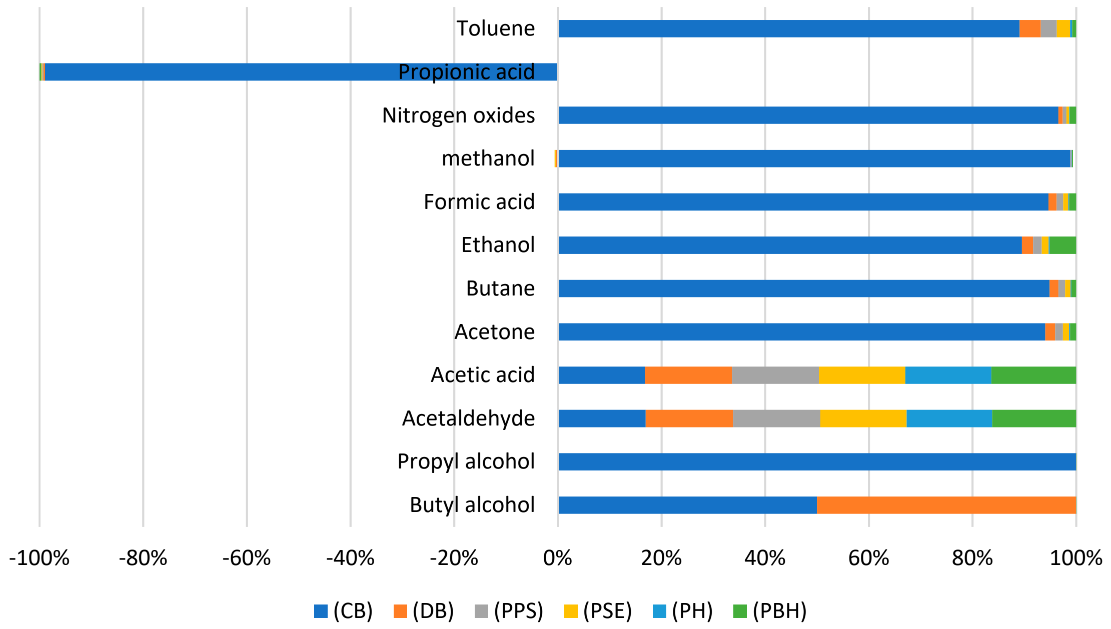

Figure S9. The results of the characterization of the environmental consequences for the ozone formation category affecting the ecosystem obtained as a result of the PLA bottle shaping process (own study).

Table S10. The results of the characterization of environmental consequences for the ozone layer depletion category obtained as a result of the PLA bottle shaping process (own study).

Substance	Unit	(CB)	(DB)	(PPS)	(PSE)	(PH)	(PBH)
butyl alcohol	DALY	1.61 × 10^{-10}	1.61 × 10^{-10}	1.6 × 10^{-10}	1.59 × 10^{-10}	1.58 × 10^{-10}	1.56 × 10^{-10}
nitric oxide	DALY	4.46 × 10^{-15}	1.83 × 10^{-17}	1.1 × 10^{-17}	1.13 × 10^{-17}	1.5 × 10^{-18}	2.01 × 10^{-17}
ethane. 1.1.1-trichloro- hcfc-140	DALY	3.78 × 10^{-14}	2.42 × 10^{-14}	1.83 × 10^{-14}	1.5 × 10^{-14}	2.5 × 10^{-15}	3.34 × 10^{-15}
ethane. 1.1.2-trichloro-1.2.2-trifluoro-. cfc-113	DALY	1.28 × 10^{-13}	4.5 × 10^{-15}	3.37 × 10^{-15}	2.79 × 10^{-15}	4.61 × 10^{-16}	7.26 × 10^{-15}
ethane. 1.2-dichloro-1.1.2.2-tetrafluoro-. cfc-114	DALY	1.25 × 10^{-15}	8.03 × 10^{-16}	6.07 × 10^{-16}	4.97 × 10^{-16}	8.29 × 10^{-17}	1.11 × 10^{-16}

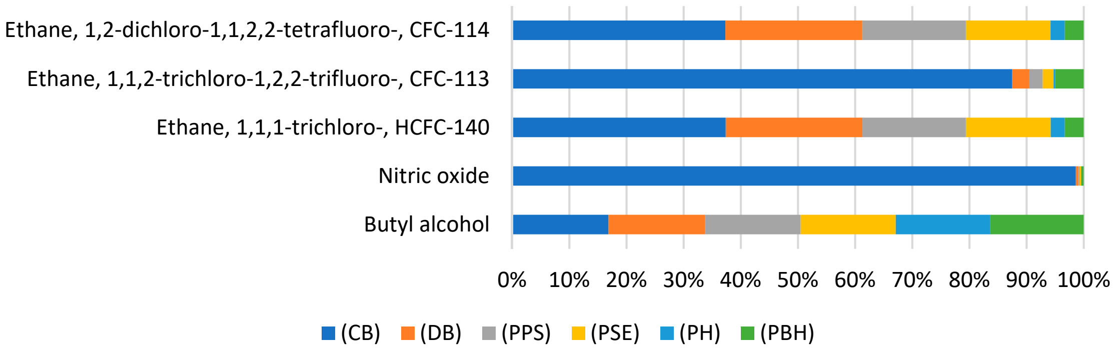

Figure S10. The results of the characterization of environmental consequences for the ozone layer depletion category obtained as a result of the PLA bottle shaping process (own study).

Table S11. Results of characterization of environmental consequences for the category of fresh water eutrophication obtained as a result of the PLA bottle shaping process (own study).

Substance	Unit	(CB)	(DB)	(PPS)	(PSE)	(PH)	(PBH)
phosphate	species.yr	5.7438×10^{-12}	5.7438×10^{-12}	6.9117×10^{-13}	5.6584×10^{-13}	9.434×10^{-14}	1.3357×10^{-13}
phosphorus	species.yr	2.7374×10^{-12}	-9.82×10^{-016}	1.03×10^{-016}	-5.86×10^{-016}	1.40×10^{-017}	2.99×10^{-016}
phosphorus	species.yr	3.40×10^{-015}	1.62×10^{-015}	1.2263×10^{-15}	1.0042×10^{-15}	1.674×10^{-16}	2.2348×10^{-16}

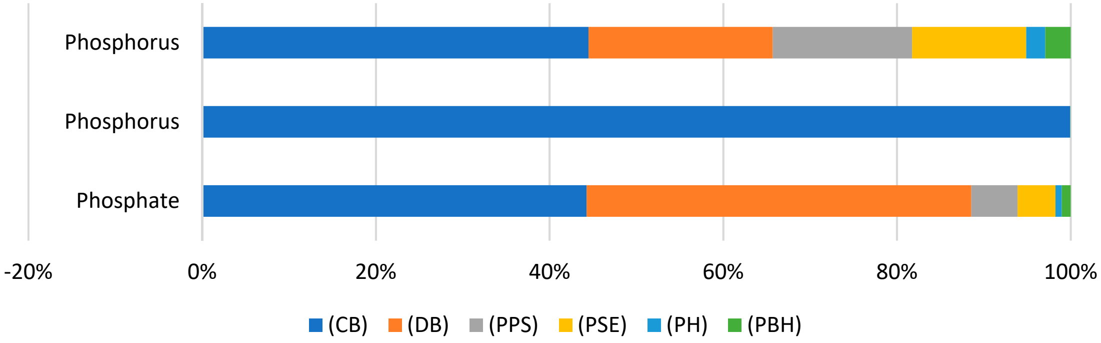

Figure S11. Results of characterization of environmental consequences for the category of fresh water eutrophication obtained as a result of the PLA bottle shaping process (own study).

Table S12. Results of the characterization of the environmental consequences for the freshwater ecotoxicity category obtained as a result of the PLA bottle shaping process (own study).

Substance	Unit	(CB)	(DB)	(PPS)	(PSE)	(PH)	(PBH)
butyl alcohol	species.yr	5.13×10^{-24}	5.97×10^{-28}	4.47×10^{-28}	3.7×10^{-28}	6.1×10^{-29}	3.74×10^{-28}
propyl alcohol	species.yr	2.81×10^{-22}	2.55×10^{-24}	2×10^{-24}	1.61×10^{-24}	2.65×10^{-25}	4.28×10^{-24}
acetaldehyde	species.yr	4.06×10^{-20}	4.05×10^{-20}	4.05×10^{-20}	4.01×10^{-20}	3.98×10^{-20}	3.91×10^{-20}

acetic acid	species.yr	3.37×10^{-21}	8.07×10^{-23}	6.12×10^{-23}	5×10^{-23}	8.35×10^{-24}	2.25×10^{-23}
acetic acid	species.yr	3.45×10^{-17}	1.04×10^{-20}	7.8×10^{-21}	6.42×10^{-21}	1.07×10^{-21}	3.41×10^{-21}
acetone	species.yr	3.58×10^{-21}	7.33×10^{-23}	5.6×10^{-23}	4.54×10^{-23}	7.64×10^{-24}	4.41×10^{-23}
acetonitrile	species.yr	3.31×10^{-21}	2.1×10^{-24}	1.59×10^{-24}	1.3×10^{-24}	2.17×10^{-25}	8.09×10^{-25}
acetyl chloride	species.yr	4.17×10^{-20}	1.25×10^{-24}	9.32×10^{-25}	7.73×10^{-25}	1.27×10^{-25}	5.7×10^{-25}
acrylic acid	species.yr	9.77×10^{-24}	-1.6×10^{-26}	9.9×10^{-27}	-9×10^{-27}	1.26×10^{-27}	2.78×10^{-26}
benzene	species.yr	2×10^{-20}	2.21×10^{-21}	1.68×10^{-21}	1.37×10^{-21}	2.29×10^{-22}	9.9×10^{-22}
cadmium	species.yr	3.85×10^{-18}	1.22×10^{-19}	9.54×10^{-20}	7.59×10^{-20}	1.3×10^{-20}	6.49×10^{-20}
carboxylic acid	species.yr	3.62×10^{-18}	3.04×10^{-22}	2.3×10^{-22}	1.89×10^{-22}	3.14×10^{-23}	1.06×10^{-22}
chloroacetic acid	species.yr	2.67×10^{-15}	$4.72 \times 10{-}20$	3.57×10^{-20}	2.92×10^{-20}	4.87×10^{-21}	1.77×10^{-20}
cobalt	species.yr	7.85×10^{-19}	2.84×10^{-20}	2.16×10^{-20}	1.76×10^{-20}	2.95×10^{-21}	1.02×10^{-20}
copper	species.yr	3.23×10^{-16}	1.04×10^{-17}	8.16×10^{-18}	6.48×10^{-18}	1.11×10^{-18}	3.27×10^{-18}
ethanol	species.yr	2.7×10^{-24}	1.11×10^{-26}	6.66×10^{-27}	6.82×10^{-27}	9.08×10^{-28}	1.22×10^{-26}
ethyl oxide	species.yr	7.44×10^{-23}	-6.7×10^{-23}	3.41×10^{-25}	-4×10^{-23}	4.65×10^{-26}	7.35×10^{-25}
formaldehyde	species.yr	4.74×10^{-18}	2.37×10^{-19}	1.8×10^{-19}	1.47×10^{-19}	2.46×10^{-20}	4.91×10^{-20}
formic acid	species.yr	6.7×10^{-19}	4.23×10^{-22}	3.22×10^{-22}	2.62×10^{-22}	4.39×10^{-23}	1.65×10^{-22}
mercury	species.yr	6.39×10^{-20}	2.25×10^{-21}	1.71×10^{-21}	1.4×10^{-21}	2.34×10^{-22}	6.51×10^{-22}
methanol	species.yr	3×10^{-20}	3×10^{-20}	2.99×10^{-20}	2.98×10^{-20}	2.97×10^{-20}	2.94×10^{-20}
methyl acetate	species.yr	3.67×10^{-24}	3.67×10^{-24}	3.67×10^{-24}	3.66×10^{-24}	3.66×10^{-24}	3.64×10^{-24}
molybdenum	species.yr	3.53×10^{-19}	8.95×10^{-21}	7.02×10^{-21}	5.55×10^{-21}	9.58×10^{-22}	3.52×10^{-21}
nickel	species.yr	8.99×10^{-17}	3.47×10^{-18}	2.91×10^{-18}	2.16×10^{-18}	3.96×10^{-19}	1.22×10^{-18}

propionic acid	species.yr	5.94 × 10^{-23}	2.21 × 10^{-25}	1.67 × 10^{-25}	1.37 × 10^{-25}	2.29 × 10^{-26}	9.04 × 10^{-26}
propylene oxide	species.yr	6.26 × 10^{-22}	6.09 × 10^{-22}	6.08 × 10^{-22}	6.04 × 10^{-22}	6.01 × 10^{-22}	5.95 × 10^{-22}
selenium	species.yr	4.33 × 10^{-18}	1 × 10^{-18}	7.59 × 10^{-19}	6.22 × 10^{-19}	1.04 × 10^{-19}	1.29 × 10^{-19}
silver	species.yr	1.15 × 10^{-18}	3.65 × 10^{-22}	3.72 × 10^{-22}	2.29 × 10^{-22}	5.06 × 10^{-23}	3.77 × 10^{-22}
sodium formate	species.yr	7.29 × 10^{-24}	7.1 × 10^{-24}	7.09 × 10^{-24}	7.03 × 10^{-24}	6.99 × 10^{-24}	6.89 × 10^{-24}
styrene	species.yr	8.29 × 10^{-24}	-2 × 10^{-24}	5.46 × 10^{-26}	-1.2 × 10^{-24}	7.37 × 10^{-27}	9.66 × 10^{-26}
sulphuric acid	species.yr	1.35 × 10^{-19}	3.5 × 10^{-22}	3.99 × 10^{-22}	2.22 × 10^{-22}	5.41 × 10^{-23}	4.95 × 10^{-21}
sulphuric acid	species.yr	7.3 × 10^{-20}	4.71 × 10^{-22}	3.65 × 10^{-22}	2.92 × 10^{-22}	4.97 × 10^{-23}	2.01 × 10^{-22}
xylene	species.yr	3.57 × 10^{-21}	8.51 × 10^{-22}	6.43 × 10^{-22}	5.27 × 10^{-22}	8.77 × 10^{-23}	1.16 × 10^{-22}

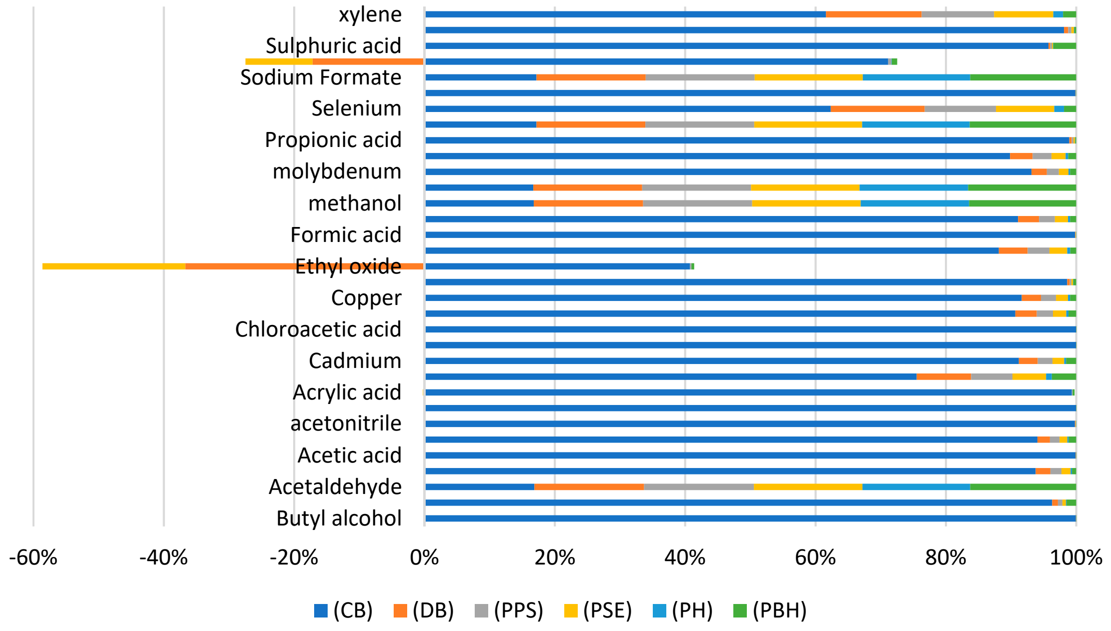

Figure S12. Results of the characterization of the environmental consequences for the freshwater ecotoxicity category obtained as a result of the PLA bottle shaping process (own study).

© 2020 by the authors. Licensee MDPI, Basel, Switzerland. This article is an open access article distributed under the terms and conditions of the Creative Commons Attribution (CC BY) license (http://creativecommons.org/licenses/by/4.0/).
